# Spillover and pandemic properties of zoonotic viruses with high host plasticity

**DOI:** 10.1038/srep14830

**Published:** 2015-10-07

**Authors:** Christine Kreuder Johnson, Peta L. Hitchens, Tierra Smiley Evans, Tracey Goldstein, Kate Thomas, Andrew Clements, Damien O. Joly, Nathan D. Wolfe, Peter Daszak, William B. Karesh, Jonna K. Mazet

**Affiliations:** 1 One Health Institute, School of Veterinary Medicine, University of California, Davis, CA USA; 2 USAID, Bureau for Global Health, Washington DC, USA; 3 Metabiota, San Francisco, CA USA; 4 EcoHealth Alliance, New York, NY USA

## Abstract

Most human infectious diseases, especially recently emerging pathogens, originate from animals, and ongoing disease transmission from animals to people presents a significant global health burden. Recognition of the epidemiologic circumstances involved in zoonotic spillover, amplification, and spread of diseases is essential for prioritizing surveillance and predicting future disease emergence risk. We examine the animal hosts and transmission mechanisms involved in spillover of zoonotic viruses to date, and discover that viruses with high host plasticity (i.e. taxonomically and ecologically diverse host range) were more likely to amplify viral spillover by secondary human-to-human transmission and have broader geographic spread. Viruses transmitted to humans during practices that facilitate mixing of diverse animal species had significantly higher host plasticity. Our findings suggest that animal-to-human spillover of new viruses that are capable of infecting diverse host species signal emerging disease events with higher pandemic potential in that these viruses are more likely to amplify by human-to-human transmission with spread on a global scale.

Emerging, re-emerging, and endemic zoonotic diseases continue to place a substantial burden on global health, particularly where dense human populations and pressures on environmental and economic resources are greatest. Over one billion cases of human zoonotic disease are estimated to occur annually, and novel emerging zoonoses have resulted in hundreds of billions of dollars in economic losses^1^. Given the rich diversity of animal life on our planet, it is not surprising that animals are the source of most human infectious diseases, with centuries of intimate contact with domesticated species facilitating the early transmission of the most adaptable pathogens to humans^2^. Recent recognition that the majority of emerging infectious disease events have wildlife origins^3^ highlights the need for a deep understanding of the type of contact between wild animals and people that enables disease transmission. Opportunities for close contact between humans and wild animals are relatively rare compared to contact with domestic animals, yet recent emergence of many diseases, such as severe acute respiratory syndrome, Nipah virus encephalitis, and Ebola, highlight the threat that wildlife pathogens pose to global health security^4^.

After centuries of documented outbreaks, we have now begun to unravel the mechanisms underlying disease transmission from animals to people. Here, we focus on zoonotic viruses, which are the most frequently emerging human pathogen, constituting less than 15% of all known species of human pathogens, but over 65% of pathogens discovered since 1980^5^. We seek to understand the mechanisms facilitating transmission of viruses from animals to people, with special attention to the human activities enabling direct and indirect contact with wild animal hosts resulting in recent human outbreaks. By evaluating data reported for all known zoonotic viruses, we test long-held assumptions regarding common traits among viruses that have spilled over from animals and activities facilitating their transmission. We use network analyses to evaluate sharing of viruses by animal hosts and high-risk transmission interfaces involving wildlife, and we use regression modeling to identify human activities linked to key pandemic properties among viruses including viral sharing among taxonomically diverse hosts, amplification by human-to-human transmission, and international spread (Fig. 1). Our findings uncover key transmission mechanisms involved in zoonotic virus emergence to inform global disease surveillance and preventive measures needed to mitigate zoonotic threats.

## Results

Through systematic evaluation of data reported in the scientific literature on zoonotic viruses, we identify several key virus characteristics and transmission mechanisms that are synergistic to zoonotic virus spillover, amplification by human-to-human transmission, and global spread. The majority (94%) of zoonotic viruses described to date (n = 162) are RNA viruses, which is 28 times higher (95% CI 13.9–62.5, exact P < 0.001) than the proportion of RNA viruses among all vertebrate viruses recognized, indicating that RNA viruses are far more likely to be zoonotic than DNA viruses, as has been reported among human pathogens^6^. Epidemiological circumstances involved in recent zoonotic transmission from animals to people are summarized here for 95 viruses with data on human activities enabling direct and indirect contact disease transmission and animal host taxa implicated in transmission. In general, wild animals were suggested as the source of zoonotic transmission for 91% (86/95) of zoonotic viruses compared to 34% (32/95) of viruses transmitted from domestic animals, and 25% (24/95) with transmission described from both wild and domestic animals (see Supplementary Table). Wild animals, which include a taxonomically diverse range of thousands of species, were significantly more likely to be a source for animal-to-human spillover of viruses than domesticated species (exact P = 0.001). Wild rodents were implicated as a source of spillover for 58% (55/95) of zoonotic viruses, particularly for zoonotic arenaviruses (n = 8/8, exact P = 0.019) and zoonotic bunyaviruses (n = 20/24, exact P = 0.004). Primates were implicated as a source of zoonotic retroviruses (exact P = 0.017), while bats were more implicated for zoonotic paramyxoviruses (exact P = 0.011) and most zoonotic rhabdoviruses (6/8, exact P = 0.002).

**Figure 2:**
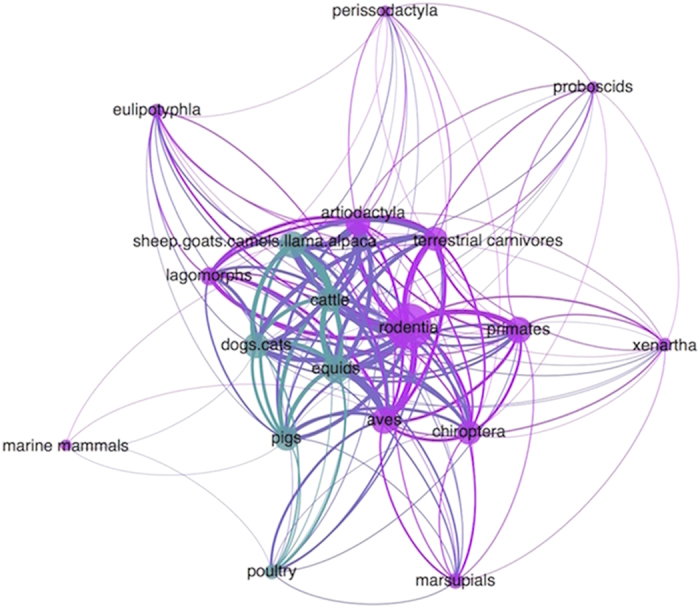
Host unipartite network map showing high host plasticity among zoonotic viruses with wild and domestic animal hosts connected by shared viruses. High connectivity between hosts by more shared viruses is evident for domestic animal hosts (green) and wild animal hosts (purple) that are most centrally located. Host node size is proportionate to the number of connections each host has to another host based on shared viruses. The width of each edge connecting hosts is relative to the number of viruses shared by the connection between hosts.

Emerging pathogens have been noted for their ability to infect a range of animal hosts^5^,^7^,^8^,^9^,^10^. We find that most (63%) zoonotic viruses infecting humans were reported in animal hosts from at least two different taxonomic orders, and 45% were reported in four or more orders, in addition to humans. The virus-host unipartite network illustrates high connectivity among host groups sharing zoonotic viruses and the central role domestic animals play in cross-species transmission (Fig. 2). In a Poisson model predicting host range and evaluating common hosts and high-risk transmission interfaces, viruses with domestic animal hosts occurred in twice as many host orders than other viruses (Table 1). Most domestic animal groups clustered in the middle of the host network with high centrality measures and a high number of shared viruses (Fig. 2), indicating that domestic animals play a key role in cross-species transmission of zoonotic viruses. Among viruses from wildlife, we found higher host plasticity (ie, hosts from a higher number of taxonomic orders) in viruses transmitted at high-risk interfaces involving wild animals kept as pets, maintained in sanctuaries or zoos, and sold at markets, which were collapsed into one category due to similar effect and significance in the final Poisson model. We also found that vector-borne viruses were reported in three times the number of host taxonomic groups than non-vector-borne viruses, indicating that vector-borne pathogens have significantly broader host range than non-vector-borne viruses.

**Figure 3:**
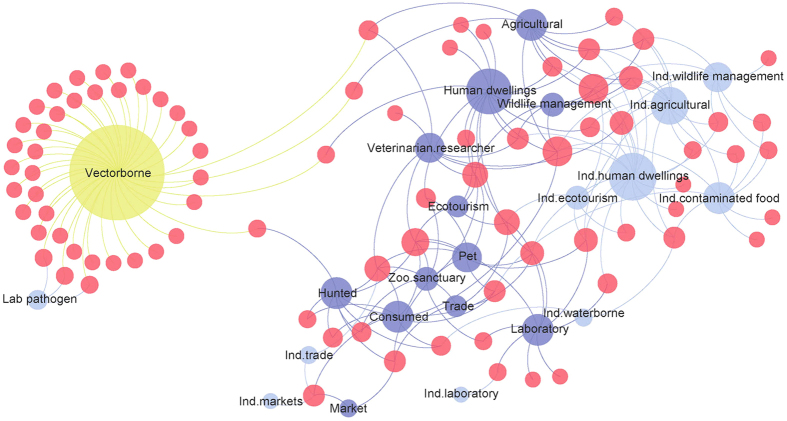
Epidemiologic bipartite network map showing high-risk disease transmission interfaces shared by zoonotic viruses transmitted from wildlife to humans. High-risk interfaces are shown with node size proportionate to the number of viruses reported for each transmission interface, categorized according to (1) direct contact with wildlife (dark blue), (2) indirect contact with wildlife (light blue), and (3) transmission by vector (yellow). Virus node size (red, n = 86 viruses) reflects the number of connections to different transmission interfaces and ecological plasticity of viruses through use of multiple transmission opportunities. Highly connected and more central interfaces facilitated transmission of more viruses, providing an epidemiologic picture of circumstances likely to promote future disease emergence, and important targets for disease surveillance and preventive measures.

Based on data published to date, transmission of zoonotic viruses to humans occurs by direct or indirect contact with wildlife in a diverse array of interconnected animal-to-human interfaces, with little overlap with viruses transmitted primarily by vectors (Fig. 3). Zoonotic virus spillover from wildlife was most frequent in and around human dwellings and in agricultural fields, as well as at interfaces with occupational exposure to animals (hunters, laboratory workers, veterinarians, researchers, wildlife management, zoo and sanctuary staff). Primate hosts were most frequently cited as the source of viruses transmitted by direct contact during hunting (exact P = 0.051) and in laboratories (exact P = 0.009), while rodent hosts were more likely to be implicated in transmission by indirect contact in and around human dwellings (exact P < 0.001) and in agricultural fields (exact P = 0.001). Approximately 40% of zoonotic viruses involving wild animals required arthropod vectors for transmission to humans, with vectors providing an effective bridge for transmission of diseases from wild animals that do not normally contact humans. Zoonotic viruses with wild avian hosts were most likely to involve vectors (exact P < 0.001). Network analysis of disease transmission from wild animals illustrates that vector-borne viruses were the least connected to other transmission interfaces (Fig. 3), consistent with effective control of vector-borne diseases by elimination of vectors or contact with vectors. In contrast, 22% of viruses transmitted from domestic animals to humans were by vector only, with close proximity interactions with domestic animals enabling direct pathogen transmission to humans.

Once animal viruses have spilled over into humans, human-to-human transmission of zoonoses facilitates sustained spread of disease with a rapidity and reach infeasible for zoonotic viruses requiring contact with animal hosts for each transmission opportunity. Human-to-human transmissibility was described for 20% of zoonotic viruses investigated here (Supplementary Table). We find virus host plasticity to be positively correlated with capability for human-to-human transmission (Table 1). In a logistic regression model predicting virus capability for human-to-human transmission, we find viruses were significantly more likely to be human-to-human transmissible with each increase in virus host plasticity (count of host orders and ecological groups). Furthermore, we find viruses in the arenaviridae and filoviridae families to be more likely to possess human-to-human transmissibility, along with viruses transmitted by direct contact with hunted and consumed wildlife (Table 1). Hunting poses special risk for cross-species disease transmission of blood-borne zoonotic viruses^11^,^12^ as evidenced by re-emerging threats, including ebolaviruses^13^ and primate retroviruses^14^,^15^,^16^. Our findings therefore support speculation that hunting of high-risk host species carries an increased probability of spillover of zoonotic viruses that can be further spread by human-to-human transmission^13^.

We further characterized zoonotic virus capacity for spread by categorizing viruses according to geographic range in a single country (16%), >1 country in 1–3 World Health Organization-defined (WHO) regions (55%), or ≥4 WHO regions (29%), and used ordinal logistic regression to evaluate characteristics of viruses in broader range categories. We find viruses were more likely to be in broader geographic range categories with increasing host plasticity (Table 1). Among all high risk interfaces and hosts, only viruses transmitted to humans by contact with wild animals in the wildlife trade and in laboratories, such as lymphocytic choriomeningitis virus^17^, monkeypox virus^18^, herpes B virus^19^, and Marburg^20^, were more likely to have broader geographic reach.

## Discussion

Wild animals were implicated as a source of disease spillover to humans for the vast majority of zoonotic viruses, further substantiating the concept that the diversity of wildlife on our planet has provided a rich pool of viruses, a fraction of which have successfully adapted to infect humans. Our findings indicate that high viral host plasticity is an important trait that is predictive of pandemic potential of viruses in the zoonotic pool, not only because wide host range was common among viruses that have spilled over from animals to humans, but also because this trait was associated with increased human-to-human transmission and spread on a global scale. Reporting bias must be considered in the interpretation of any association based on data reported in the literature, and the relationship between human-to-human transmissibility and host plasticity could be biased by increased research effort for viruses that have been shown to be transmissible among humans. However our analyses identified a strong linear relationship between host plasticity and likelihood of human-to-human transmissibility, and we estimate zoonotic viruses found in 10 host orders are 12 times more likely to be human-to-human transmissible than zoonotic viruses found in only one animal host order. Human-to-human transmission of viruses with high host plasticity is consistent with the hypothesis that evolutionary selection for viruses with greater ability to adapt rapidly to new hosts co-selects for viruses capable of effective intraspecies transmission in the new host. Evolutionary selection of viruses capable of infecting a wide range of hosts has been a key hypothesis underpinning disease emergence theory^7^,^21^, and we provide evidence for the importance of viral host plasticity as a synergistic trait aiding mechanisms of disease transmission, particularly at the high-risk human-animal interfaces reported here.

Human practices facilitating heightened contact between taxonomically diverse animal hosts has likely facilitated selection of viruses with high host plasticity and sharing of zoonotic diseases. Zoonotic viruses reported in domestic animals had a significantly wider host range than viruses not shared by domesticate species. Increased research effort targeting diseases in domesticated species could bias data towards this finding, but we also detected increased host range among viruses transmitted by wildlife kept in similarly confined circumstances. Increased host plasticity among viruses shared by domestic animals supports the concept that the breeding and keeping of taxonomically diverse domesticated species in regular close contact with people for centuries has enabled evolutionary adaptive selection for mutation-prone RNA viruses capable of cross-species transmission^2^. For the many viruses shared by wildlife and domestic animals, domesticated species play a critical role in facilitating direct contact with people, as well as amplification of disease transmission in intensive animal production facilities.

Our finding of significantly higher host plasticity among viruses transmitted by direct contact with wildlife kept as pets or in zoos and sanctuaries provides additional evidence to support the premise that confining taxonomically diverse species in close proximity selects for transmission of adaptable viruses with high host plasticity, even among wildlife. Diverse species of wild animals that are confined in zoos, sanctuaries, kept as pets, and sold at markets are also subject to circumstances that facilitate cross-species virus transmission via intimate contact, particularly for zoonotic viruses already adapted to transmission among domesticated animals. Vectorborne transmission similarly enables opportunities for effective contact across diverse animal hosts, which is consistent with our finding of higher host plasticity among vectorborne viruses. Through this mechanism, vector-borne transmission has facilitated emergence of animal diseases in humans, particularly those from wildlife, and, for viruses with generalist vectors, this transmission route is an effective method for interspecific dispersal^6^.

Here we provide an epidemiologic picture of the animal-human transmission networks likely to perpetuate future disease emergence, and our findings add to previous efforts to guide global health research geographically^3^. In addition to an emphasis on vector control, the myriad of other high-risk interfaces with human activities that have facilitated animal-to-human viral spillover should be a focus for education and interventions directed at disease prevention. More in depth investigation of the epidemiology of zoonoses at high risk human-animal interfaces is needed to assess risk of viral disease emergence and direct global, as well as local, disease prevention and control. Risk for a new human pandemic is likely highest at the high-risk interfaces facilitating disease threats in the past. Unfortunately, wild animal hosts and high-risk interfaces facilitating spillover of zoonotic viruses, particularly beyond their first emergence, remains vastly under-reported. Adequate data on circumstances at the point of disease spillover are lacking for many viruses because animal involvement in zoonotic disease exposure is very difficult to ascertain and this information is often not linked to diagnoses in published reports. Global animal disease data are largely incomplete due to inadequate livestock and wildlife health surveillance worldwide. Resulting ascertainment biases are especially problematic for spillover events that do not involve professions likely to self-report, as is likely the case for veterinarians, researchers, and scientists working at laboratory facilities. Detailed patient histories that elucidate activities precipitating animal exposure will greatly assist in completing the epidemiologic picture underlying the emergence of many zoonotic viruses. This, together with heightened surveillance to gather data on human practices enabling contact with animals in settings with diverse host assemblages, particularly at high-risk interfaces under-reported to date, will direct us towards critical control points for disease control and behavior change interventions aimed at prevention.

## Methods

### Zoonotic Virus Datasets

Peer-reviewed scientific literature was searched for reports on zoonotic viruses transmitted from animals to humans using the Web of Science electronic library database for published reports through 2010. An initial list of zoonotic pathogens was established with database searches for topic keywords (zoonotic, zoonoses, and infectious animal disease, emerging wildlife disease) and cross checked with World Health Organization (WHO), Food and Agricultural Organization (FAO), Centers for Disease Control and Prevention (CDC), and the World Organization for Animal Health (OIE) web-based reports, and previously published compilations of human infectious diseases and human emerging infectious disease events^3^,^22^. Individual pathogen-specific searches using the Web of Science database were then made using pathogen common and scientific names to identify general transmission properties and specific circumstances involved in disease transmission from animals to humans reported in the peer-reviewed literature. Among 162 zoonotic viruses, data on animal hosts and human activities associated with naturally occurring animal-to-human transmission from 1990–2010 were collated and summarized for each virus. Viral family categories and virus genome characteristic (RNA vs DNA) was compiled using the National Center for Biotechnology Information^23^.

Zoonotic viruses were included in analyses of interfaces and hosts if data were available on the circumstances surrounding virus transmission from animals to humans from 1990–2010 in scientific reports searched as described above (n = 95 viruses, Supplementary Table). Viral transmission from animals to humans was determined as documented infection or seroconversion, without regard to disease severity. General transmission categories were used to summarize disease transmission by i) direct or indirect contact with wild animals, ii) transmission from direct or indirect contact with domestic animals, iii) transmission by vector involving a wildlife host, domestic animal host, or both. Each virus was also categorized as human-to-human transmissible if horizontal human-to-human transmission was reported, as for transmission from animals to humans (by documented infection or seroconversion, without regard to disease severity) based on search of all reports for each virus in the scientific literature. For this study, human-to-human transmission excluded transmission between humans by vectors.

For all viruses transmitted from wildlife, data on circumstances of transmission were collated from all reports for each virus to identify interfaces that best described the human activities suspected or confirmed to enable effective contact and natural (ie non-experimental) transmission of zoonotic viruses to people. Transmission interfaces involving wildlife were stratified by direct and indirect contact transmission and summarized in categories describing human contact as follows i) wild animals in and around human dwellings, ii) wild animals hunted, iii) wild animals consumed, iv) wild animals kept as pets, v) wild animals housed in laboratories, vi) wild animals sold in markets, vii) wild animals kept in zoos and sanctuaries, viii) wild animal exposure during agricultural activities, ix) wild animal exposure during ecotourism activities, x) wild animal exposure during wildlife management activities in protected areas, xi) virus exposure in laboratory settings (lab pathogen), and xii) virus exposure via contaminated water.

For all viruses included in analyses, an extended search was conducted to identify confirmed or suspected hosts serving as a source of spillover as reported in the peer-reviewed literature based on virus detection by molecular assay, serological assay, or virus isolation. Animal species included were implicated in the scientific reports as hosts suspected in animal-to-human transmission of a given virus, either through direct contact, indirect contact or vector-borne transmission. Host species were then classified a priori according to ecological circumstances for human contact (domesticated species, wild terrestrial species, and wild marine mammal species), which we expected to modify any potential host-pathogen phylogenetic relationships based on purely taxonomic classification. Stratification of animal host categories according to general circumstances of human contact was also important so that analyses could inform on risk interfaces and intervention strategies. Wild terrestrial host species were then categorized further by taxonomic order, except for orders within the superorder xenarthra, which were collapsed into one category (n = 6 zoonotic viruses). Marine mammal orders were also combined due to sparse data, as marine mammals were implicated in spillover of only 3 zoonotic viruses. Due to the large number of viruses reported in domesticated animals, domestic species were grouped according to taxonomy and stratified by similarity in circumstances for human use of animals and their products; i) cattle, ii) equids, iii) goats, sheep, llamas, alpaca, camels, iv) pigs, v) poultry (chickens, ducks, geese), and vi) dogs and cats. Virus host range (host plasticity) was calculated as the total count of animal taxonomic orders and ecological groups recognized as hosts involved in animal-to-human spillover for each virus.

A literature search was similarly conducted to identify geographic range reported for each virus in humans and animals. Geographic distribution in animals and people encompassed importation to another country by infected persons or animals if secondary amplification by animal-to-human or human-to-human transmission occurred. Zoonotic viruses were further classified according to 3 categories of international spread based on published reports as to whether viruses had been reported within 1) a single country only, 2) more than one country but only 1–3 WHO regions, or 3) more than one country and ≥4 WHO regions^24^ (Supplementary Table).

While search effort was standardized for all viruses in our approach to the literature review, viruses varied in the number of scientific reports available describing their traits, hosts, and geographic range. All virus, host species, and interface data were summarized as binomial variables for each individual virus, in order to account for a variable number of reports and documented spillover events per virus, and adjust for likely increased research effort for viruses that infect humans and domesticated species. Each virus was designated as the unit of analysis for which we compared viral traits, animal hosts involved in spillover, and human activities noted at the point of spillover.

### Statistics

Virus genome category (RNA vs DNA) was compared between zoonotic viruses (n = 162) and all viruses reported to infect humans and other vertebrates minus the zoonotic viruses (n = 956) using Fisher’s exact test. Bipartite affiliation (two-mode) networks were generated for virus-host and virus-interface matrix data to evaluate connectedness between host orders and between high-risk disease transmission interfaces involving wildlife. Betweenness centrality was measured for all viruses to indicate the number of connections with wild and domestic animal hosts in the virus-host network, along with the centrality of each virus within the host network, relative to all other zoonotic viruses. Betweenness centrality for each virus was calculated as the number of geodesic paths that pass through a node, standardized by the total number of virus nodes in the network, multiplied by 100. A unipartite (one-mode) network was generated to illustrate host taxonomic orders and groups connected by shared viruses. Network analyses were conducted in the network analysis platform Gephi, using the force-directed algorithm ForceAtlas2 to generate a virus-interface network display^25^. Centrality indices were normalised for two-mode data^26^ using specialized software for social network analysis (UCINET 6 for Windows).

Unadjusted bivariate relationships between viral family, interface categories, and host taxa were examined using exact statistics. Viral traits and transmission circumstances were further evaluated for multivariable associations with virus host plasticity using Poisson regression to evaluate the influence of putative viral traits and high-risk interfaces on the count of host taxonomic orders and ecological groups reported for each virus. Factors evaluated for their relationship with host range included viral family, general transmission category involving domestic animals, wild animals, or vectors, and specific direct and indirect contact wildlife transmission interfaces. Incidence rate (indicating count of host orders) ratios were estimated for all significant independent factors associated with virus host range in the Poisson model (P < 0.05). Viral traits, general transmission categories, wildlife transmission interfaces, and virus host plasticity measures were similarly evaluated for associations with virus capability for human-to-human transmission. Virus host plasticity, general transmission categories, and wildlife transmission interfaces were also evaluated for associations with international spread using ordered logistic regression adjusting for clustering of random effects within virus family.

For all multivariable models, putative risk factors with P < 0.20 in univariable analyses were entered forward stepwise and retained in models if P < 0.05. Correlated variables were not included in the same model but deviance measures were used to evaluate changes in model fit to the data with each parameter independently. In all models, variables with <3 categories were evaluated for difference in magnitude, direction, and significance of effect between categories using the likelihood ratio statistic and similar categories were collapsed. Overall model fit was evaluated using Hosmer-Lemeshow goodness-of-fit test and measures of information criteria. Incidence rate and odds ratios were estimated with 95% confidence intervals. Univariable and multivariable statistical analyses were conducted using STATA 13.1 SE (College Station, TX, USA).

## Additional Information

**How to cite this article**: Johnson, C.K. *et al.* Spillover and pandemic properties of zoonotic viruses with high host plasticity. *Sci. Rep.* **5**, 14830; doi: 10.1038/srep14830 (2015).

## Supplementary Material

Supplementary Information

## Figures and Tables

**Figure 1:**
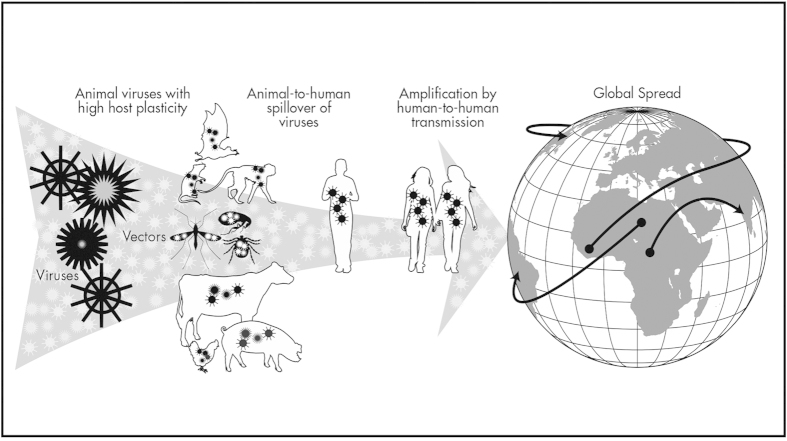
Pandemic properties of zoonotic viruses that spill over from animals to humans and spread by secondary transmission among humans. Key characteristics of pandemic potential that were evaluated for associations with viral traits and high-risk disease transmission interfaces include host plasticity, human-to-human transmissibility, and geographic distribution. Human practices that promote transmission of mutation-prone RNA viruses able to infect a wide range of taxonomically diverse hosts, including wild and domestic animals, act synergistically to facilitate viral emergence, particularly for viruses capable of human-to-human transmission and broad geographic spread (map and illustration created using Adobe Illustrator CS6).

**Table 1:**
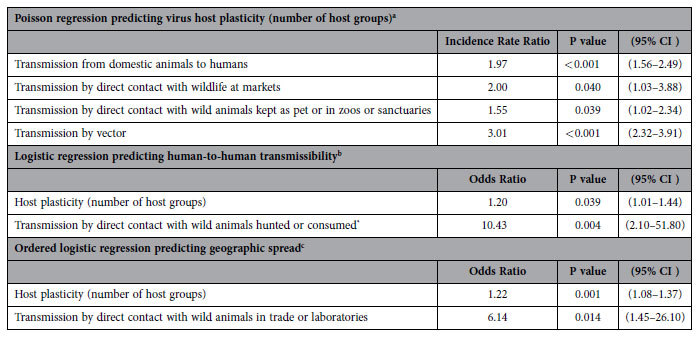
Host and epidemiologic correlates of zoonotic virus emergence.

Multivariable regression models with viral traits and transmission interfaces significantly associated with zoonotic virus host plasticity, human-to-human transmissibility, and geographic spread.

^a^Viral family was included as a main effect in the model. Viral families significantly related to number of host orders were reovirus (IRR = 2.07 (1.21–3.55), P = 0.008), rhabdovirus (IRR = 1.59 (1.13–2.24), P = 0.008), and a collapsed virus family group with bornavirus and hepatitis E virus (IRR = 4.48 (2.77–7.25), P < 0.001).

^b^Viral family was included as a main effect in the model. Viral families with a significantly higher probability of human-to-human transmission were arenavirus (OR = 62.6 (8.09–485), P < 0.001) and filovirus (OR = 52.92 (3.90–719), P = 0.003).

^c^Virus family was included as a random effect using robust standard error estimation clustered on virus family.

^*^High-risk disease transmission interface categories ‘hunting’ and ‘consumed’ were similar in their association with virus capability for human-to human transmission so these categories were collapsed for this model.
