# Supplementary material for: Spillover and pandemic properties of zoonotic viruses with high host plasticity
Source: Sci Rep. 2015 Oct 7;5:14830. doi: 10.1038/srep14830 (PMC4595845; doi:10.1038/srep14830)
Supplement: Supplementary Information [file srep14830-s1.doc]

**Title:** Spillover and pandemic properties of zoonotic viruses with high host plasticity

Christine Kreuder Johnson^1^, Peta L. Hitchens^1^, Tierra Smiley Evans^1^, Tracey Goldstein^1^, Kate Thomas^1^, Andrew Clements^2^, Damien O. Joly^3^, Nathan D. Wolfe^3^, Peter Daszak^4^, William B. Karesh^4^, Jonna K. Mazet^1^

**Supplemental Information**

Supplementary Table: Zoonotic viruses with summarized host and transmission interface data (n=95). General transmission categories were grouped here to show disease transmission from direct or indirect contact with wild animals (wild), transmission from direct or indirect contact with domestic animals (domestic), transmission by vector involving wildlife host (wild by vector), transmission by vector involving domestic animal host (domestic by vector), and transmission by vector with either wild and/or domestic animal hosts (by vector). Recognized human-to-human transmissibility, virus host range among wild and domestic animal taxonomic orders, and host plasticity measures (betweenness centrality calculated in the virus-host network) are also shown.

|  | **Zoonotic Virus** | **Family** | **General Transmission Category** | **Host Range**  (# host orders) | **Host Plasticity** (betweenness  centrality) | **Human to Human Transmissible** | **Geographic Spread Category** | **References** |
| --- | --- | --- | --- | --- | --- | --- | --- | --- |
|  | Alkhurma hemorrhagic fever virus | Flaviviridae | wild & domestic by vector | 1 | 0.00000 | no | 2 | 1–7 |
|  | Andes virus | Bunyaviridae | wild | 1 | 0.00000 | yes | 2 | 8–23 |
|  | Australian bat lyssavirus | Rhabdoviridae | wild | 2 | 0.05426 | no | 2 | 24–39 |
|  | Barmah forest virus | Togaviridae | wild & domestic by vector | 3 | 0.11170 | no | 1 | 40–46 |
|  | Bermejo virus | Bunyaviridae | wild | 1 | 0.00000 | no | 2 | 21,47 |
|  | Bhanja virus | Bunyaviridae | wild & domestic by vector | 11 | 2.26170 | no | 3 | 48–53 |
|  | Borna disease virus | Bornaviridae | wild, domestic | 12 | 3.76271 | yes | 3 | 54–64 |
|  | Bovine papular stomatitis virus | Poxviridae | domestic | 2 | 0.00974 | no | 3 | 65–69 |
|  | Buffalopox virus | Poxviridae | domestic | 1 | 0.00000 | no | 2 | 69–74 |
|  | Bundibugyo ebolavirus | Filoviridae | wild | 0 | 0.00000 | yes | 1 | 75–78 |
|  | Cache Valley virus | Bunyaviridae | wild by vector | 9 | 1.33894 | no | 2 | 79–91 |
|  | Chandipura virus | Rhabdoviridae | wild, wild by vector | 4 | 0.17489 | no | 1 | 92–95 |
|  | Chapare virus | Arenaviridae | wild | 1 | 0.00000 | no | 1 | 96–98 |
|  | Chikungunya virus | Togaviridae | wild by vector | 4 | 0.87557 | no | 3 | 53,99–121 |
|  | Choclo virus | Bunyaviridae | wild | 1 | 0.00000 | no | 1 | 21,122,123 |
|  | Colorado tick fever virus | Reoviridae | wild & domestic by vector | 4 | 0.27461 | no | 2 | 124–128 |
|  | Cowpox | Poxviridae | wild, domestic | 9 | 2.79031 | no | 2 | 69,129–141 |
|  | Crimean Congo hemorrhagic fever virus | Bunyaviridae | wild, domestic, & by vector | 9 | 1.27243 | yes | 3 | 142–168 |
|  | Dengue fever | Flaviviridae | wild by vector | 1 | 0.00000 | no | 3 | 101–103,121,169–193 |
|  | Dobrava virus | Bunyaviridae | wild | 1 | 0.00000 | no | 2 | 194–207 |
|  | Duvenhage virus | Rhabdoviridae | wild | 1 | 0.00000 | no | 2 | 33,35,38,208–211 |
|  | Eastern equine encephalitis virus | Togaviridae | wild by vector | 7 | 0.93908 | no | 2 | 99,212–219 |
|  | Edge Hill virus | Flaviviridae | wild by vector | 2 | 0.02592 | no | 1 | 220,221 |
|  | Encephalomyocarditis virus | Picornaviridae | wild, domestic | 4 | 0.79878 | no | 3 | 222–226 |
|  | European bat lyssavirus type 1 | Rhabdoviridae | wild | 4 | 0.23293 | no | 2 | 227–240 |
|  | European bat lyssavirus type 2 | Rhabdoviridae | wild | 1 | 0.00000 | no | 2 | 227–234,238,241,242 |
|  | Everglades virus | Togaviridae | wild by vector | 4 | 0.39571 | no | 1 | 243–245 |
|  | Getah virus | Togaviridae | domestic, domestic by vector | 5 | 0.19205 | no | 2 | 99,246–251 |
|  | Guanarito virus | Arenaviridae | wild | 1 | 0.00000 | yes | 1 | 98,252–258 |
|  | Guaroa virus | Bunyaviridae | wild by vector | 3 | 0.34038 | no | 2 | 172,259 |
|  | Hantaan virus | Bunyaviridae | wild | 1 | 0.00000 | no | 3 | 20–23,194,260–268 |
|  | Hendra virus | Paramyxoviridae | domestic | 2 | 0.03744 | no | 2 | 34,269–279 |
|  | Hepatitis E | Hepeviridae | wild, domestic | 12 | 3.12934 | yes | 3 | 280–287 |
|  | Herpes B | Herpesviridae | wild | 1 | 0.00000 | no | 3 | 288–299 |
|  | Ilheus virus | Flaviviridae | wild by vector | 9 | 3.14787 | no | 2 | 300,301 |
|  | Influenza A | Orthomyxoviridae | wild, domestic | 6 | 5.52025 | yes | 3 | 302–309 |
|  | Influenza B | Orthomyxoviridae | wild | 1 | 0.00000 | yes | 3 | 310,311 |
|  | Jamestown Canyon virus | Bunyaviridae | wild by vector | 4 | 0.10008 | no | 2 | 86,212,312–320 |
|  | Japanese encephalitis | Flaviviridae | wild by vector | 4 | 0.18939 | no | 3 | 94,321–326 |
|  | Junin virus | Arenaviridae | wild | 1 | 0.00000 | yes | 1 | 253,256,327–336 |
|  | Juquitiba-like virus | Bunyaviridae | wild | 1 | 0.00000 | no | 2 | 337,338 |
|  | Kairi virus | Bunyaviridae | wild by vector | 2 | 0.09737 | no | 2 | 79,339,340 |
|  | Kyasunur Forest disease virus | Flaviviridae | wild by vector | 6 | 1.53682 | no | 2 | 3–6,169,341–343 |
|  | La Crosse virus | Bunyaviridae | wild by vector | 4 | 0.27461 | no | 1 | 86,124,312,344–351 |
|  | Laguna Negra virus | Bunyaviridae | wild | 1 | 0.00000 | no | 2 | 13,16,20–22,337,352–358 |
|  | Lassa virus | Arenaviridae | wild | 1 | 0.00000 | yes | 3 | 327,359–375 |
|  | Ljungan virus | Picornaviridae | wild | 3 | 0.49656 | no | 2 | 376–379 |
|  | Lujo virus | Arenaviridae | wild | 1 | 0.00000 | yes | 2 | 380,381 |
|  | Lymphocytic choriomeningitis virus | Arenaviridae | wild, domestic | 2 | 0.26899 | no | 3 | 382–392 |
|  | Machupo virus | Arenaviridae | wild | 1 | 0.00000 | yes | 1 | 98,253,254,393,394 |
|  | Marburg virus | Filoviridae | wild | 2 | 0.12741 | yes | 2 | 395–411 |
|  | Mayaro virus | Togaviridae | wild by vector | 6 | 1.38672 | no | 2 | 99,172,412–418 |
|  | Mokola virus | Rhabdoviridae | domestic | 4 | 0.43558 | no | 2 | 33,35,38,209,242,419–422 |
|  | Monkeypox virus | Poxviridae | wild | 4 | 0.75450 | yes | 3 | 69,423–436 |
|  | Monongahela virus | Bunyaviridae | wild | 1 | 0.00000 | no | 1 | 437,438 |
|  | Muju virus | Bunyaviridae | wild | 1 | 0.00000 | no | 1 | 260,438 |
|  | Murray Valley encephalitis virus | Flaviviridae | wild by vector | 6 | 0.30960 | no | 2 | 439–443 |
|  | Nipah virus | Paramyxoviridae | wild, domestic | 3 | 0.12393 | yes | 2 | 34,269,277–279,444–468 |
|  | Omsk hemorrhagic fever virus | Flaviviridae | wild, domestic, & by vector | 4 | 0.30372 | no | 1 | 4,469–471 |
|  | O'nyong nyong virus | Togaviridae | wild by vector | 0 | 0.00000 | no | 2 | 100,472–475 |
|  | Orf virus | Poxviridae | domestic | 4 | 0.09315 | no | 3 | 476–492 |
|  | Oropouche virus | Bunyaviridae | wild by vector | 1 | 0.00000 | no | 2 | 493–495 |
|  | Powassan virus | Flaviviridae | wild by vector | 2 | 0.08332 | no | 2 | 4,124,496–500 |
|  | Pseudocowpox virus | Poxviridae | domestic | 3 | 0.02827 | no | 3 | 501–504 |
|  | Puumala virus | Bunyaviridae | wild | 2 | 0.08332 | no | 2 | 21–23,194,196,438,505–521 |
|  | Rabies virus | Rhabdoviridae | wild, domestic | 10 | 2.01739 | no | 3 | 35,242,522–532 |
|  | Reston ebola virus | Filoviridae | wild, domestic | 2 | 0.08516 | no | 2 | 533–536 |
|  | Rift Valley fever virus | Bunyaviridae | wild, domestic, & by vector | 9 | 2.35230 | no | 2 | 537–546 |
|  | Ross River virus | Togaviridae | wild & domestic by vector | 11 | 2.44876 | no | 2 | 42,99,547–552 |
|  | Rotavirus A | Reoviridae | wild, domestic | 10 | 2.87215 | yes | 3 | 553–566 |
|  | Sandfly fever virus | Bunyaviridae | domestic by vector | 5 | 0.23979 | no | 3 | 22,53,142,537,567–576 |
|  | SARS coronavirus | Coronaviridae | wild | 5 | 0.31665 | yes | 3 | 444,577–582 |
|  | Sealpox | Poxviridae | wild | 1 | 0.00000 | no | 2 | 583–587 |
|  | Seoul virus | Bunyaviridae | wild | 1 | 0.00000 | no | 3 | 20–23,194,260,265,337,588–596 |
|  | Simian foamy virus | Retroviridae | wild | 1 | 0.00000 | no | 2 | 597–605 |
|  | Simian immunodeficiency virus | Retroviridae | wild | 1 | 0.00000 | no | 2 | 606–613 |
|  | Simian retrovirus type D | Retroviridae | wild | 1 | 0.00000 | no | 2 | 614–618 |
|  | Sin Nombre virus | Bunyaviridae | wild | 1 | 0.00000 | no | 2 | 8,9,12,21–23,100,101,194,438,619–634 |
|  | Sindbis virus | Togaviridae | wild by vector | 5 | 0.25821 | no | 3 | 46,52,99,101,121,142,475,635–643 |
|  | St. Louis encephalitis virus | Flaviviridae | wild by vector | 6 | 1.42191 | no | 2 | 124,213,644–655 |
|  | Sudan ebola virus | Filoviridae | unknown | 0 | 0.00000 | yes | 2 | 76,656–661 |
|  | Tahyna virus | Bunyaviridae | wild by vector | 4 | 0.26590 | no | 3 | 52,121,340,475,643,662–667 |
|  | Tick borne encephalitis | Flaviviridae | wild, wild by vector | 6 | 0.71747 | no | 2 | 142,169,594,668–683 |
|  | Tioman virus | Paramyxoviridae | wild, domestic | 2 | 0.04547 | no | 2 | 34,269,667,684,685 |
|  | Tula virus | Bunyaviridae | wild | 1 | 0.00000 | no | 2 | 22,23,686–689 |
|  | Usutu virus | Flaviviridae | wild by vector | 3 | 0.17841 | no | 2 | 121,340,635,643,687,690–703 |
|  | Venezualan equine encephalitis | Togaviridae | wild & domestic by vector | 5 | 0.56228 | no | 2 | 170,172,243,244,252,704–712 |
|  | Vesicular stomatitis | Rhabdoviridae | domestic, & by vector | 14 | 4.63961 | no | 2 | 252,713–722 |
|  | Wesselsbron virus | Flaviviridae | domestic, & by vector | 5 | 0.18756 | no | 2 | 722–725 |
|  | West Nile virus | Flaviviridae | domestic, & by vector | 6 | 0.66653 | no | 3 | 38,52,121,142,210,313,316,475,635,643,666,667,690,726–732 |
|  | Western equine encephalitis virus | Togaviridae | wild by vector | 5 | 0.51692 | no | 2 | 99,212,213,654,655,733,734 |
|  | Whitewater Arroyo virus | Arenaviridae | wild | 1 | 0.00000 | yes | 2 | 98,329,735–741 |
|  | Yellow fever virus | Flaviviridae | wild by vector | 1 | 0.00000 | no | 3 | 102,103,121,172,740,742–746 |
|  | Zaire ebola virus | Filoviridae | wild, domestic | 5 | 0.62028 | yes | 2 | 657,661,747–754 |
|  | Zika virus | Flaviviridae | wild by vector | 7 | 1.75492 | no | 3 | 76,755–761 |

Supplementary References

1. Charrel, R. N. *et al.* Low diversity of Alkhurma hemorrhagic fever virus, Saudi Arabia, 1994-1999. *Emerg. Infect. Dis.* **11,** 683–8 (2005).

2. Madani, T. A. *et al.* Alkhumra (Alkhurma) virus outbreak in Najran, Saudi Arabia: epidemiological, clinical, and laboratory characteristics. *J. Infect.* **62,** 67–76 (2011).

3. Charrel, R. N. *et al.* Alkhurma hemorrhagic fever virus in *Ornithodoros savignyi* ticks. *Emerg. Infect. Dis.* **13,** 153–5 (2007).

4. Charrel, R. N. *et al.* Complete coding sequence of the Alkhurma virus, a tick-borne Flavivirus causing severe hemorrhagic fever in humans in Saudi Arabia. *Biochem. Biophys. Res. Commun.* **287,** 455–61 (2001).

5. Alzahrani, A. G. *et al.* Alkhurma hemorrhagic fever in humans, Najran, Saudi Arabia. *Emerg. Infect. Dis.* **16,** 1882–1887 (2010).

6. Carletti, F. *et al.* Alkhurma hemorrhagic fever in travelers returning from Egypt, 2010. *Emerg. Infect. Dis.* **16,** 1979–1983 (2010).

7. Memish, Z. A., Charrel, R. N., Zaki, A. M. & Fagbo, S. F. Alkhurma haemorrhagic fever - a viral haemorrhagic disease unique to the Arabian Peninsula. *Int. J. Antimicrob. Agents* **36 Suppl 1,** S53–7 (2010).

8. Lázaro, M. E. *et al.* Clusters of Hantavirus infection, southern Argentina. *Emerg. Infect. Dis.* **13,** 104–10 (2007).

9. Torres-Pérez, F., Palma, R. E., Hjelle, B., Ferrés, M. & Cook, J. A. Andes virus infections in the rodent reservoir and in humans vary across contrasting landscapes in Chile. *Infect. Genet. Evol.* **10,** 820–25 (2010).

10. Medina, R. A. *et al.* Ecology, genetic diversity, and phylogeographic structure of Andes virus in humans and rodents in Chile. *J. Virol.* **83,** 2446–59 (2009).

11. Galeno, H. *et al.* First human isolate of Hantavirus (Andes virus) in the Americas. *Emerg. Infect. Dis.* **8,** 657–661 (2002).

12. Gonzalez Della Valle, M. *et al.* Andes virus associated with Hantavirus pulmonary syndrome in northern Argentina and determination of the precise site of infection. *Am. J. Trop. Med. Hyg.* **66,** 713–20 (2002).

13. Padula, P. J. *et al.* Andes virus and first case report Bermejo virus causing fatal pulmonary syndrom. *Emerg. Infect. Dis.* **8,** 437–439 (2002).

14. Castillo, C. *et al.* Prevalence of antibodies to Hantavirus among family and health care worker contacts of persons with Hantavirus Cardiopulmonary syndrome: lack of evidence for nosocomial transmission of Andes virus to health care workers in Chile. *Am. J. Trop. Med. Hyg.* **70,** 302–4 (2004).

15. Castillo, C., Naranjo, J., Sepúlveda, A., Ossa, G. & Levy, H. Hantavirus pulmonary syndrom due to Andes virus in Temuco, Chile; clinical experience with 16 adults. *Clin. Investig. Crit. Care* **120,** 548–554 (2001).

16. Chu, Y.-K., Owen, R. D., Gonzalez, L. M. & Jonsson, C. B. The complex ecology of Hantavirus in Paraguay. *Am. J. Trop. Med. Hyg.* **69,** 263–8 (2003).

17. Riquelme, R. *et al.* Hantavirus pulmonary syndrome, southern Chile. *Emerg. Infect. Dis.* **9,** 1438–1443 (2003).

18. Martinez, V. P. *et al.* Hantavirus pulmonary syndrome in Argentina, 1995-2008. *Braz. J. Infect. Dis.* **16,** 1853–1860 (2010).

19. Pini, N. *et al.* Hantavirus infection in humans and rodents, Northwestern Argentina. *Emerg. Infect. Dis.* **9,** 1070–1076 (2003).

20. Kariwa, H., Yoshimatsu, K. & Arikawa, J. Hantavirus infection in east Asia. *Comp. Immunol. Microbiol. Infect. Dis.* **30,** 341–56 (2007).

21. Nelson, R. *et al.* Confirmation of Choclo virus as the cause of Hantavirus Cardiopulmonary syndrome and high serum antibody prevalence in Panama. *Hournal Med. Virol.* **82,** 1586–1593 (2010).

22. Richter, M. H., Hanson, J. D., Cajimat, M. N., Milazzo, M. L. & Fulhorst, C. F. Geographical range of Rio Mamoré virus (family Bunyaviridae, genus Hantavirus) in association with the small-eared pygmy rice rat *(Oligoryzomys microtis)*. *Vector borne zoonotic Dis.* **10,** 613–20 (2010).

23. Schmaljohn, C. & Hjelle, B. Hantaviruses: a global disease problem. *Emerg. Infect. Dis.* **3,** 95–104 (1997).

24. Fielding, J. & Nayda, C. Postexposure prophylaxis for Australian bat lyssavirus in south Australia, 1996 to 2003. *Aust. Vet. J.* **83,** 233–234 (2005).

25. Guyatt, K. J. *et al.* A molecular epidemiological study of Australian bat lyssavirus. *J. Gen. Virol.* **84,** 485–496 (2003).

26. Warrilow, D. *et al.* Public health surveillance for Australian bat lyssavirus, in Queensland, Australia, 2000–-2001. *Emerg. Infect. Dis.* **9,** 262–264 (2003).

27. McColl, K. A. *et al.* Pathogenesis studies with Australian bat lyssavirus in grey-headed flying foxes *(Pteropus poliocephalus)*. *Aust. Vet. J.* **80,** 636–41 (2002).

28. Warrilow, D., Smith, I. L., Harrower, B. & Smith, G. A. Sequence analysis of an isolate from a fatal human infection of Australian bat lyssavirus. *Virology* **297,** 109–119 (2002).

29. McCall, B. J. *et al.* Potential exposure to Australian bat lyssavirus, Queensland, 1996-1999. *Emerg. Infect. Dis.* **6,** 259–64 (2000).

30. Arguin, P. M. *et al.* Serologic evidence of Lyssavirus infections among bats, the Philippines. *Emerg. Infect. Dis.* **8,** 258–262 (2002).

31. Reynes, J. *et al.* Serologic evidence of Lyssavirus infection in bats, Cambodia. *Emerg. Infect. Dis.* **10,** 2231–2234 (2004).

32. Lumlertdacha, B. *et al.* Survey for bat Lyssaviruses, Thailand. *Emerg. Infect. Dis.* **11,** 232–6 (2005).

33. Kuzmin, I. V *et al.* Shimoni bat virus, a new representative of the Lyssavirus genus. *Virus Res.* **149,** 197–210 (2010).

34. Iehlé, C. *et al.* Henipavirus and Tioman virus antibodies in Pteropodid bats, Madagascar. *Emerg. Infect. Dis.* **13,** 159–162 (2007).

35. Arai, Y. T., Kuzmin, I. V., Kameoka, Y. & Botvinkin, A. D. New Lyssavirus genotype from the lesser mouse-eared bat *(Myotis blythi)*, Kyrghzstan. *Emerg. Infect. Dis.* **9,** 333–337 (2003).

36. Kuzmin, I. *et al.* Lyssavirus surveillance in bats, Bangladesh. *Emerg. Infect. Dis.* **12,** 486–488 (2006).

37. Kuzmin, I. *et al.* Bat Lyssaviruses (Aravan and Khujand) from Central Asia: phylogenetic relationships according to N, P and G gene sequences. *Virus Res.* **97,** 65–79 (2003).

38. Kuzmin, I. V., Hughes, G. J., Botvinkin, A. D., Orciari, L. A. & Rupprecht, C. E. Phylogenetic relationships of Irkut and West Caucasian bat viruses within the Lyssavirus genus and suggested quantitative criteria based on the N gene sequence for Lyssavirus genotype definition. *Virus Res.* **111,** 28–43 (2005).

39. Harris, S. L. *et al.* European bat Lyssaviruses: distribution, prevalence, and implications for conservation. *Biol. Conserv.* **131,** 193–210 (2006).

40. Naish, S. *et al.* Socio-environmental predictors of Barmah Forest virus transmission in coastal areas, Queensland, Australia. *Trop. Med. Int. Heal.* **14,** 247–56 (2009).

41. Jacups, S. P., Whelan, P. I. & Currie, B. J. Ross River virus and Barmah Forest virus infections: a review of history, ecology, and predictive models, with implications for tropical northern Australia. *Vector-Borne Zoonotic Dis.* **8,** 283–97 (2008).

42. Jeffery, J. A. L., Kay, B. H. & Ryan, P. A. Role of *Verrallina funerea* (Diptera : Culicidae) in transmission of Barmah Forest virus and Ross River virus in coastal areas of eastern Australia. *J. Med. Entomol.* **43,** 1239–1247 (2006).

43. Quinn, A. H. E., Gatton, M. L., Hall, G., Young, M. & Ryan, P. A. Analysis of Barmah Forest virus disease activity in Queensland, Australia, 1993 – 2003: identification of a large, isolated outbreak of disease. *J. Med. Entomol.* **42,** 882–890 (2005).

44. Jeffery, J. A., Ryan, P. A., Lyons, S. A., Thomas, P. T. & Kay, B. H. Spatial distribution of vectors of Ross River virus and Barmah Forest virus on Russell Island, Moreton Bay, Queensland. *Aust. J. Entomol.* **41,** 329–338 (2002).

45. Bi, P., Tong, S., Donald, K., Parton, K. & Hobbs, J. Short reports Southern Oscillation Index and transmission of the Barmah Forest virus infection in Queensland, Australia. *J. Epidemiol. Community Health* **54,** 69–70 (2000).

46. Johansen, C. A., Mackenzie, J. S., Smith, D. W. & Lindsay, M. D. A. Prevalence of neutralising antibodies to Barmah Forest, Sindbis and Trubanaman viruses in animals and humans in the south-west of Western Australia. *Aust. J. Zool.* **53,** 51 (2005).

47. Padula, P. J. *et al.* Andes virus and First Case Report Bermejo Virus Causing Fatal Pulmonary Syndrom. *Emerg. Infect. Dis.* **8,** 437–439 (2002).

48. Sikutová, S. *et al.* Serological survey of domestic animals for Tick-borne Encephalitis and Bhanja viruses in northeastern Hungary. *Vet. Microbiol.* **135,** 267–71 (2009).

49. Hubálek, Z. Biogeography of tick-borne Bhanja virus (Bunyaviridae) in Europe. *Interdiscip. Perspect. Infect. Dis.* **2009,** 372691 (2009).

50. Sang, R. *et al.* Tickborne arbovirus surveillance in market livestock, Nairobi, Kenya. *Emerg. Infect. Dis.* **12,** 1074–80 (2006).

51. Hubalek, Z. Geographic distribution of Bhanja virus. *Folia Parasitol. (Praha).* **34,** 77–86 (1987).

52. Bárdos, V. *et al.* Prevalence of arbovirus antibodies in sera of animals in Sri Lanka. *Bull. World Health Organ.* **61,** 987–90 (1983).

53. Madic, J., Huber, D. & Lugovic, B. Serologic survey for selected viral and rickettsial agents of brown bears *(Ursus arctos)* in Croatia. *J. Wildl. Dis.* **29,** 372–376 (1993).

54. Richt, J. A. & Rott, R. Borna disease virus: a mystery as an emerging zoonotic pathogen. *Vet. J.* **161,** 24–40 (2001).

55. Puorger, M. E. *et al.* Distribution of Borna disease virus antigen and RNA in tissues of naturally infected bicolored white-toothed shrews, *Crocidura leucodon*, supporting their role as reservoir host species. *Vet. Pathol.* **47,** 236–44 (2010).

56. Kinnunen, P. M. *et al.* Serological evidence for Borna disease virus infection in humans, wild rodents and other vertebrates in Finland. *J. Clin. Virol.* **38,** 64–9 (2007).

57. Dürrwald, R., Kolodziejek, J., Muluneh, A., Herzog, S. & Nowotny, N. Epidemiological pattern of classical Borna disease and regional genetic clustering of Borna disease viruses point towards the existence of to-date unknown endemic reservoir host populations. *Microbes Infect.* **8,** 917–29 (2006).

58. Wensman, J. J., Berg, M. & Berg, A.-L. Experiences of Borna disease virus infection in Sweden. *Acta Pathol. Microbiol. Immunol. Scand. Suppl.* **116,** 46–9 (2008).

59. Jordan, I. & Lipkin, W. Borna disease virus. *Rev. Med. Virol.* (2001).

60. Selten, J., Vliet, K. van & Pleyte, W. Borna disease virus and schizophrenia in Surinamese immigrants to the Netherlands. *Med. Microbiol. Immunol* (2000).

61. Chalmers, R., Thomas, D. & Salmon, R. Borna disease virus and the evidence for human pathogenicity: a systematic review. *QJM* **98,** 255–274 (2005).

62. Kamhieh, S. & Flower, R. Borna disease virus (BDV) infection in cats a concise review based on current knowledge. *Vet. Q.* **28,** 65–73 (2006).

63. Vahlenkamp, T. & Konrath, A. Persistence of Borna disease virus in naturally infected sheep. *J. Virol.* **76,** 9735–9743 (2002).

64. Richt, J., Herzog, S., Haberzettl, K. & Rott, R. Demonstration of Borna disease virus-specific RNA in secretions of naturally infected horses by the polymerase chain reaction. *Med. Microbiol. Immunol.* **182,** 293–304 (1993).

65. Leonard, D. *et al.* Unusual Bovine Papular Stomatitis virus infection in a British dairy cow. *Vet. Rec.* **164,** 65 (2009).

66. Inoshima, Y., Nakane, T. & Sentsui, H. Severe dermatitis on cattle teats caused by Bovine Papular Stomatitis virus. *Vet. Rec.* **164,** 311–2 (2009).

67. Knezevic, M., Jovanovic, M. & Asanin, R. Bovine papular stomatitis - Its first occurrence in Serbia. *Acta Vet. Brno.* **50,** 147–154 (2000).

68. Aguilar-Setién, A., Correa-Girón, P., Hernández-Baumgarten, E., Cruz-Gómez, A. & Hernandez-Jauregui, P. Bovine papular stomatitis, first report of the disease in Mexico. *Cornell Vet.* **70,** 10–8 (1980).

69. Essbauer, S., Pfeffer, M. & Meyer, H. Zoonotic Poxviruses. *Vet. Microbiol.* **140,** 229–36 (2010).

70. Singh, R. K. *et al.* Buffalopox: an emerging and re-emerging zoonosis. *Anim. Health Res. Rev.* **8,** 105–14 (2007).

71. Zafar, A. *et al.* P1707 Report of multicentre outbreak of Buffalopox virus infection in burn units, Karachi, Pakistan. *Int. J. Antimicrob. Agents* **29,** S484 (2007).

72. Yadav, S., Hosamani, M., Balamurugan, V., Bhanuprakash, V. & Singh, R. K. Partial genetic characterization of viruses isolated from pox-like infection in cattle and buffaloes: evidence of buffalo pox virus circulation in Indian cows. *Arch. Virol.* **155,** 255–61 (2010).

73. Bhanuprakash, V. *et al.* Zoonotic infections of buffalopox in India. *Zoonoses Public Health* **57,** e149–55 (2010).

74. Venkatesan, G. *et al.* Emerging and re-emerging zoonotic buffalopox infection: a severe outbreak in Kolhapur (Maharashtra), India. *Vet. Ital.* **46,** 439–48 (2010).

75. MacNeil, A. *et al.* Proportion of deaths and clinical features in Bundibugyo ebola virus infection, Uganda. *Emerg. Infect. Dis.* **16,** 1969–1973 (2010).

76. Wamala, J. F. *et al.* Ebola hemorrhagic fever associated with novel virus strain , Uganda, 2007-2008. *Emerg. Infect. Dis.* **16,** 2007–2008 (2010).

77. Towner, J. S. *et al.* Newly discovered ebola virus associated with hemorrhagic fever outbreak in Uganda. *PLoS Pathog.* **4,** e1000212 (2008).

78. Alsop, Z. Ebola outbreak in Uganda “atypical”, say experts. *Lancet* **370,** 2085–2085 (2007).

79. Tauro, L. B., Almeida, F. L. & Contigiani, M. S. First detection of human infection by Cache Valley and Kairi viruses (Orthobunyavirus) in Argentina. *Trans. R. Soc. Trop. Med. Hyg.* **103,** 197–9 (2009).

80. Blackmore, C. G. & Grimstad, P. R. Cache Valley and Potosi viruses (Bunyaviridae) in white-tailed deer *(Odocoileus virginianus)*: experimental infections and antibody prevalence in natural populations. *Am. J. Trop. Med. Hyg.* **59,** 704–9 (1998).

81. Sexton, D. J. *et al.* Life-threatening Cache Valley virus infection. *N. Engl. J. Med.* **336,** 547–549 (1997).

82. McLean, R. G., Calisher, C. H. & Parham, G. L. Isolation of Cache Valley virus and detection of antibody for selected arboviruses in Michigan horses in 1980. *Am. J. Vet. Res.* **48,** 1039–41 (1987).

83. Calisher, C. H. *et al.* Distribution of Bunyamwera serogroup viruses in North America, 1956-1984. *Am. J. Trop. Med. Hyg.* **35,** 429–43 (1986).

84. Aguirre, A. A., Mclean, R. G., Cook, R. S. & Quan, T. J. Serologic survey for selected arboviruses and other potential pathogens in wildlife from Mexico. *J. Wildl. Dis.* **28,** 435–442 (1992).

85. Miller, D. S. *et al.* Serologic survey for selected infectious disease agents in Swift and Kit foxes from the western United States. *J. Wildl. Dis.* **36,** 798–805 (2000).

86. Neitzel, D. F. & Grimstad, P. R. Serological evidence of Califonia group and Cache Valley virus infection in Minnesota white-tailed deer. *J. Wildl. Dis.* **27,** 230–237 (1991).

87. Sahu, S. P. *et al.* Serologic survey of cattle in the northeastern and north central United States, Virginia, Alaska, and Hawaii for antibodies to Cache Valley and antigenically related viruses (Bunyamwera serogroup virus). *Am. J. Trop. Med. Hyg.* **67,** 119–22 (2002).

88. Wang, H., Nattanmai, S., Kramer, L. D., Bernard, K. a & Tavakoli, N. P. A duplex real-time reverse transcriptase polymerase chain reaction assay for the detection of California serogroup and Cache Valley viruses. *Diagn. Microbiol. Infect. Dis.* **65,** 150–7 (2009).

89. Blackmore, C. G. M. & Grimstad, P. R. Evaluation of the Eastern Cottontail *Sylvilagus floridanus* as an amplifying vertebrate host for Cache Valley virus (Bunyaviridae) in Indiana. *J. Wildl. Dis.* **44,** 188–92 (2008).

90. Campbell, G. L. *et al.* Second human case of Cache Valley virus disease. *Emerg. Infect. Dis.* **12,** 854–6 (2006).

91. Concha-Bermejillo, A. de la. Cache Valley virus is a cause of fetal malformation and pregnancy loss in sheep. *Small Rumin. Res.* **49,** 1–9 (2003).

92. Gurav, Y. K. *et al.* Chandipura virus encephalitis outbreak among children in Nagpur division, Maharashtra, 2007. *Indian J. Med. Res.* **132,** 395–9 (2010).

93. Pradesh, A. *et al.* Chandipura virus: A major cause of acute encephalitis in children in North Telangana, Andhra Pradesh, Indina. *J. Med. Virol.* **80,** 118–124 (2008).

94. Chadha, M. S. *et al.* An outbreak of Chandipura virus encephalitis in the eastern districts of Gujarat state, India. *Am. J. Trop. Med. Hyg.* **73,** 566–70 (2005).

95. Geevarghese, G. & Arankalle, V. Detection of Chandipura virus from sand flies in the genus Sergentomyia (Diptera: Phlebotomidae) at Karimnagar district, Andhra Pradesh, India. *J. Med. …* (2005).

96. Cajimat, M. *et al.* Genetic diversity among Bolivian arenaviruses. *Virus Res.* 24–31 (2009).

97. Delgado, S. *et al.* Chapare virus, a newly discovered arenavirus isolated from a fatal hemorrhagic fever case in Bolivia. *PLoS Pathog.* **4,** e1000047 (2008).

98. Charrel, R. N. & de Lamballerie, X. Zoonotic aspects of arenavirus infections. *Vet. Microbiol.* **140,** 213–20 (2010).

99. Zhai, Y. *et al.* Complete sequence characterization of isolates of Getah virus (genus Alphavirus, family Togaviridae) from China. *J. Gen. Virol.* **89,** 1446–56 (2008).

100. Powers, A. M., Brault, A. C., Tesh, R. B. & Weaver, S. C. Re-emergence of Chikungunya and O’nyong-nyong viruses: evidence for distinct geographical lineages and distant evolutionary relationships. *J. Gen. Virol.* **81,** 471–9 (2000).

101. Eisenhut, M., Schwarz, T. F. & Hegenscheid, B. Seroprevalence of Dengue, Chikungunya and Sindbis virus infections in German aid workers. *Infection* **27,** 82–5 (1999).

102. Jentes, E. S. *et al.* Acute arboviral infections in Guinea, West Africa, 2006. *Am. J. Trop. Med. Hyg.* **83,** 388–94 (2010).

103. Kuniholm, M. H. *et al.* Seroprevalence and distribution of Flaviviridae, Togaviridae, and Bunyaviridae arboviral infections in rural Cameroonian adults. *Am. J. Trop. Med. Hyg.* **74,** 1078–83 (2006).

104. Soumahoro, M.-K. *et al.* Imported Chikungunya virus infection. *Emerg. Infect. Dis.* **16,** 162–163 (2010).

105. Ayu, S. M. *et al.* Seroprevalence survey of Chikungunya virus in Bagan Panchor, Malaysia. *Am. J. Trop. Med. Hyg.* **83,** 1245–8 (2010).

106. Manimunda, S. P. *et al.* Outbreak of Chikungunya fever, Dakshina Kannada district, South India, 2008. *Am. J. Trop. Med. Hyg.* **83,** 751–4 (2010).

107. Rianthavorn, P., Prianantathavorn, K., Wuttirattanakowit, N., Theamboonlers, A. & Poovorawan, Y. An outbreak of Chikungunya in southern Thailand from 2008 to 2009 caused by African strains with A226V mutation. *Int. J. Infect. Dis.* **14,** e161–5 (2010).

108. Gaibani, P. *et al.* Serological update of the Chikungunya epidemic outbreak in Italy. *Int. J. Infect. Dis.* **14,** e209–e209 (2010).

109. Dwibedi, B. *et al.* Emergence of Chikungunya virus infection in Orissa, India. *Vector borne zoonotic Dis.* **10,** 347–54 (2010).

110. Thiboutot, M. M. *et al.* Chikungunya: a potentially emerging epidemic? *PLoS Negl. Trop. Dis.* **4,** e623 (2010).

111. Hapuarachchi, H. C. *et al.* Re-emergence of Chikungunya virus in South-east Asia: virological evidence from Sri Lanka and Singapore. *J. Gen. Virol.* **91,** 1067–76 (2010).

112. Abraham, A. M. & Sridharan, G. Chikungunya virus infection a resugent scourge. *Indian J. Med. Res.* **126,** 502–504 (2007).

113. Demanou, M. *et al.* Chikungunya outbreak in a rural area of Western Cameroon in 2006: A retrospective serological and entomological survey. *BMC Res. Notes* **3,** 128 (2010).

114. Gasque, P. *et al.* Emergence and clinical insights into the pathology of Chikungunya virus. *Expert Rev. Anti-infective Ther.* **8,** 987–996 (2010).

115. Nero, C. Chikungunya, the traveling virus. *Clin. Microbiol. Newsl.* **30,** 97–100 (2008).

116. Powers, A. M. & Logue, C. H. Changing patterns of Chikungunya virus: re-emergence of a zoonotic arbovirus. *J. Gen. Virol.* **88,** 2363–77 (2007).

117. AbuBakar, S. *et al.* Reemergence of endemic Chikungunya Malaysia. *Emerg. Infect. Dis.* **13,** 147–149 (2007).

118. Bonilauri, P. *et al.* Chikungunya Virus in *Aedes albopictus*, Italy. *Emerg. Infect. Dis.* **14,** 852–853 (2008).

119. Chahar, H. S. *et al.* Co-infections with Chikungunya virus and Dengue virus in Delhi India. *Emerg. Infect. Dis.* **15,** 1077–1080 (2009).

120. Das, T. *et al.* Chikungunya fever: CNS infection and pathologies of a re-emerging arbovirus. *Prog. Neurobiol.* **91,** 121–9 (2010).

121. Hubálek, Z. Mosquito-borne viruses in Europe. *Parasitol. Res.* **103 Suppl,** S29–43 (2008).

122. Armien, B. *et al.* High seroprevalence of hantavirus infection on the Azuero peninsula of Panama. *Am. J. Trop. Med. Hyg.* **70,** 682–7 (2004).

123. Armién, A. G. *et al.* Hantavirus infection and habitat associations among rodent populations in agroecosystems of Panama: implications for human disease risk. *Am. J. Trop. Med. Hyg.* **81,** 59–66 (2009).

124. Alatoom, A. & Payne, D. An Overview of Arboviruses and Bunyaviruses. *Lab. Med.* **40,** 237–240 (2009).

125. Brackney, M. M. *et al.* Epidemiology of Colorado tick fever in Montana, Utah, and Wyoming, 1995-2003. *Vector-borne Zoonotic Dis.* **10,** 381–5 (2010).

126. Eisen, L., Ibarra-Juarez, L. A., Eisen, R. J. & Piesman, J. Indicators for elevated risk of human exposure to host-seeking adults of the Rocky Mountain wood tick *(Dermacentor andersoni)* in Colorado. *J. Vector Ecol.* **33,** 117–128 (2008).

127. James, A. M. *et al.* Distribution, seasonality, and hosts of the Rocky Mountain wood tick in the United States. *J. Med. Entomol.* **43,** 17–24 (2006).

128. Klasco, R. Colorado tick fever. *Med. Clin. North Am.* **86,** 435–440 (2002).

129. Hemmer, C. J. *et al.* Human cowpox virus infection acquired from a circus elephant in Germany. *Int. J. Infect. Dis.* **14,** e338–40 (2010).

130. Hazel, S. M. *et al.* A longitudinal study of an endemic disease in its wildlife reservoir: cowpox and wild rodents. *Epidemiol. Infect.* **124,** 551–62 (2000).

131. Kurth, A. *et al.* Cowpox virus outbreak in banded mongooses *(Mungos mungo)* and jaguarundis *(Herpailurus yagouaroundi)* with a time-delayed infection to humans. *PLoS One* **4,** e6883 (2009).

132. Becker, C. *et al.* Cowpox virus infection in pet rat owners: not always immediately recognized. *Dtsch. Arztebl. Int.* **106,** 329–34 (2009).

133. Vorou, R. M., Papavassiliou, V. G. & Pierroutsakos, I. N. Cowpox virus infection: an emerging health threat. *Curr. Opin. Infect. Dis.* **21,** 153–6 (2008).

134. Amer, M., El-Gharib, I., Rashed, A., Farag, F. & Emara, M. Human cowpox infection in Sharkia Governorate, Egypt. *Int. J. Dermatol.* **40,** 14–7 (2001).

135. Kurth, A. *et al.* Rat-to-elephant-to-human transmission of Cowpox virus. *Emerg. Infect. Dis.* **14,** 4–6 (2008).

136. Tryland, M. *et al.* Characteristics of four cowpox virus isolates from Norway and Sweden. *APMIS* **106,** 623–35 (1998).

137. Chantrey, J. *et al.* Cowpox: reservoir hosts and geographic range. *Epidemiol. Infect.* **122,** 455–60 (1999).

138. Wolfs, T. F. W., Wagenaar, J. A., Niesters, H. G. M. & Osterhaus, A. D. M. E. Rat-to-human transmission of cowpox infection. *Emerg. Infect. Dis.* **8,** 1495–1496 (2002).

139. Generalized, S., Pelkonen, P. M., Tarvainen, K., Hynninen, A. & Kallio, E. R. K. Cowpox with sever generalized eruption Finland. *Emerg. Infect. Dis.* **9,** 1458–1461 (2003).

140. Martina, B. E. E., Doornum, G. Van, Dorrestein, G. M., Niesters, H. G. M. & Osterhaus, A. D. M. E. Cowpox transmission from rats to monkeys, the Netherlands. *Emerg. Infect. Dis.* **12,** 1005–1007 (2006).

141. Coras, B. *et al.* Cowpox and a cat. *Lancet* **365,** 446 (2005).

142. Gould, E. A., Higgs, S., Buckley, A. & Gritsun, T. S. Potential arbovirus emergence and implications for the United Kingdom. *Emerg. Infect. Dis.* **12,** 549–55 (2006).

143. Boyd, A., Fazakerley, J. K. & Bridgen, A. Pathogenesis of Dugbe virus infection in wild-type and interferon-deficient mice. *J. Gen. Virol.* **87,** 2005–9 (2006).

144. Günaydin, N. S., Aydin, K. & Yilmaz, G. Crimean-Congo hemorrhagic fever cases in the eastern Black Sea Region of Turkey: demographic, geographic, climatic, and clinical characteristics. *Turkish J. Med. Sci.* **40,** 829–834 (2010).

145. Gunes, T. Crimean-Congo hemorrhagic fever virus in high-risk population, Turkey. *Emerg. Infect. Dis.* **15,** 461–465 (2009).

146. Sharifi-Mood, B. *et al.* Clinical and epidemiologic features of crimean-congo hemorrhagic fever among children and adolescents from southeastern Iran. *Pediatr. Infect. Dis. J.* **27,** 555–7 (2008).

147. Rai, M. A., Khanani, M. R., Warraich, H. J., Hayat, A. & Ali, S. H. Crimean-Congo hemorrhagic fever in Pakistan. *J. Med. Virol.* **80,** 1004–1006 (2008).

148. Midilli, K. *et al.* Imported Crimean-Congo hemorrhagic fever cases in Istanbul. *BMC Infect. Dis.* **7,** 54 (2007).

149. Mardani, M. *et al.* Crimean-Congo hemorrhagic fever among health care workers in Iran: a seroprevalence study in two endemic regions. *Am. J. Trop. Med. Hyg.* **76,** 443–5 (2007).

150. Ergönül, O. *et al.* Zoonotic infections among veterinarians in Turkey: Crimean-Congo hemorrhagic fever and beyond. *Int. J. Infect. Dis.* **10,** 465–9 (2006).

151. Tattevin, P., Tarantola, A., Tall, A., Nabeth, P. & Michelet, C. Imported Crimean-Congo hemorrhagic fever. *J. Clin. Microbiol.* **43,** 4905–4907 (2005).

152. Athar, M. N. *et al.* Short report: Crimean-Congo hemorrhagic fever outbreak in Rawalpindi, Pakistan, February 2002. *Am. J. Trop. Med. Hyg.* **69,** 284–7 (2003).

153. W, J. & Waterman, S. H. Crimean-Congo hemorrhagic fever in man, Republic of Georgia, 2009. *Emerg. Infect. Dis.* **16,** (2010).

154. Papa, A. *et al.* Crimean-Congo hemorrhagic fever in Albania, 2001. *Eur. J. Clin. Microbiol. Infect. Dis.* **21,** 603–6 (2002).

155. Drosten, C., Herbert, D., Emmerich, P. & Schmitz, H. Crimean-Congo hemorrhagic fever in Kosovo. *J. Clin. Microbiol.* **40,** 1122–1123 (2002).

156. Tezer, H. *et al.* Crimean-Congo hemorrhagic fever in children. *J. Clin. Virol.* **48,** 184–6 (2010).

157. Aradaib, I. E. *et al.* Nosocomial outbreak of Crimean-Congo hemorrhagic fever, Sudan. *Emerg. Infect. Dis.* **16,** 837–9 (2010).

158. Ertugrul, B. *et al.* An outbreak of Crimean-Congo hemorrhagic fever in western Anatolia, Turkey. *Int. J. Infect. Dis.* **13,** e431–6 (2009).

159. Vorou, R. M. Crimean-Congo hemorrhagic fever in southeastern Europe. *Int. J. Infect. Dis.* **13,** 659–62 (2009).

160. Sun, S. *et al.* Epidemiology and phylogenetic analysis of crimean-congo hemorrhagic fever viruses in xinjiang, china. *J. Clin. Microbiol.* **47,** 2536–43 (2009).

161. Yilmaz, G. R. *et al.* The epidemiology of Crimean-Congo hemorrhagic fever in Turkey, 2002-2007. *Int. J. Infect. Dis.* **13,** 380–6 (2009).

162. Chinikar, S., Ghiasi, S. M., Hewson, R., Moradi, M. & Haeri, A. Crimean-Congo hemorrhagic fever in Iran and neighboring countries. *J. Clin. Virol.* **47,** 110–4 (2010).

163. Swanepoel, R. *et al.* Epidemiologic and clinical features of Crimean-Congo hemorrhagic fever in southern Africa. *Amercian J. Top. Med. Hygeine* **36,** 120–132 (1987).

164. Carroll, S. a, Bird, B. H., Rollin, P. E. & Nichol, S. T. Ancient common ancestry of Crimean-Congo hemorrhagic fever virus. *Mol. Phylogenet. Evol.* **55,** 1103–10 (2010).

165. Nabeth, P. *Emerging Viruses in Human Populations*. *Emerg. Viruses Hum. Popul.* **16,** 306–331 (Elsevier Science and Techonolgy, 2007).

166. Ergönül, O. Crimean-Congo haemorrhagic fever. *Lancet Infect. Dis.* **6,** 203–14 (2006).

167. Hoogstraal, H. The epidemiology of tick-born Crimean-Congo Hemorrhagic fever in Asia, Europe, and Africa. *J. Med. Entomol.* **15,** 307–417 (1979).

168. Sang, R. *et al.* Tickborne arbovirus surveillance in market livestock, Nairobi, Kenya. *Emerg. Infect. Dis.* **12,** 1074–80 (2006).

169. Kuno, G. & Chang, G.-J. J. Full-length sequencing and genomic characterization of Bagaza, Kedougou, and Zika viruses. *Arch. Virol.* **152,** 687–96 (2007).

170. Vilcarromero, S. *et al.* Venezuelan Equine Encephalitis and 2 human deaths, Peru. *Emerg. Infect. Dis.* **16,** 553–6 (2010).

171. Medlock, J. M., Snow, K. R. & Leach, S. Possible ecology and epidemiology of medically important mosquito-borne arboviruses in Great Britain. *Epidemiol. Infect.* **135,** 466–82 (2007).

172. Forshey, B. M. *et al.* Arboviral etiologies of acute febrile illnesses in western South America, 2000-2007. *PLoS Negl. Trop. Dis.* **4,** e787 (2010).

173. Dorji, T. *et al.* Diversity and origin of Dengue virus serotypes 1, 2, and 3, Bhutan. *Emerg. Infect. Dis.* **15,** 1630–1632 (2009).

174. Usman, M., Jamal, S., Tabassum, S. & Zafran, M. Outcome of Dengue fever in adults. *Gomal J. Med. Sci.* **9,** 70–73 (2011).

175. Suaya, J. *et al.* Cost of Dengue cases in eight countries in the Americas and Asia: a prospective study. *Am. J. Trop. Med. Hyg.* **80,** 846–855 (2009).

176. Mackenzie, J., Fubler, D. & Peterson, L. Emerging Flaviviruses: the spread and resurgence of Japanese Encephalitis, West Nile and Dengue viruses. *Nat. Med. Suppl.* **10,** S98–S109 (2004).

177. Mari, R. & Peydro, R. Re-Emergence of Malaria and Dengue in Europe in Current Topics in *Tropical Medicine*. *Curr. Top. Trop. Med.* 483–512 (InTech, 2012).

178. Perret, C. *et al.* Dengue-1 virus isolation during first Dengue fever outbreak on Easter Island, Chile. *Emerg. Infect. Dis.* **9,** 1465–1467 (2003).

179. Aseffa, A. Viral diseases in Ethiopia: a review. *East Afr. Med. J.* **70,** 624–626 (1993).

180. Hayes, J. *et al.* Risk factors for infection during a Dengue-1 outbreak in Maui, Hawaii, 2001. *Trans. R. Soc. Trop. Med. Hyg.* **100,** 559–556 (2006).

181. Fagbami, A., Monath, T. & Fabiyi, A. Dengue virus infections in Nigeria: a survey for antibodies in monkeys and humans. *Trans. R. Soc. Trop. Med. Hyg.* **71,** 60–65 (1977).

182. Krippner, R. & VonLaer, G. First confirmed Dengue-1 Fever cases reported from Cameroon. *J. Travel Med.* **9,** 273–274 (2002).

183. Ruche, G. La *et al.* First two autochthonous Dengue virus infections in metropolitan France, September 2010. *Euro Surveill.* **15,** 2–6 (2010).

184. Leroy, E. *et al.* Concurrent Chikungunya and Dengue virus infections during simultaneous outbreaks, Gabon, 2007. *Emerg. Infect. Dis.* **15,** 591–593 (2009).

185. Rodier, G. *et al.* Epidemic Dengue-2 in the city of Djibouti 1991-1992. *Trans. R. Soc. Trop. Med. Hyg.* **90,** 209–217 (1996).

186. Gubler, D. & Meltzer, M. Impact of Dengue/Dengue hemorrhagic fever on the developing world. *Adv. Virus Res.* **53,** 35–70 (1999).

187. Ratsitorahina, M. *et al.* Outbreak of Dengue and Chikungunya fevers, Toamasina, Madagascar, 2006. *Emerg. Infect. Dis.* **14,** 1135–1137 (2008).

188. Rico-Hesse, R. Molecular evolution and distribution of Dengue viruses type 1 and 2 in nature. *Virology* **174,** 479–493 (1990).

189. Gubler, D. & Clark, G. Dengue/Dengue Hemorrhagic fever: the emergence of a global health problem. *Emerg. Infect. Dis.* **1,** 55–57 (1995).

190. Gubler, D. Dengue and Dengue Hemorrhagic fever. *Clin. Microbiol. Rev.* **11,** 480–496 (1998).

191. Aviles, G. *et al.* Dengue reemergence in Argentina. *Emerg. Infect. Dis.* **5,** 575–578 (1999).

192. Xie, J., Weng, Y., Shen, X. & Zhao, Z. Identification of Dengue type 1 virus isolated in Fujian province by using the reverse transcription Nest-polymerase chain reaction. *Chinese J. Zoonoses* **21,** 699–701 (2005).

193. Platt, K. *et al.* Detection of Dengue virus neutralizing antibodies in bats from Costa Rica and Ecuador. *J. Med. Entomol.* **37,** 965–967 (2000).

194. Woods, C. *et al.* Domestically acquired Seoul virus causing hemorrhagic fever with renal syndrome-Maryland, 2008. *Clin. Infect. Dis.* **49,** e109–12 (2009).

195. Dzagurova, T. K. *et al.* Molecular diagnostics of hemorrhagic fever with renal syndrome during a Dobrava virus infection outbreak in the European part of Russia. *J. Clin. Microbiol.* **47,** 4029–36 (2009).

196. Nowakowska, A., Heyman, P., Knap, J. P., Burzynski, W. & Witas, M. The first established focus of Hantavirus infection in Poland, 2007. *Ann. Agric. Environ. Med.* **16,** 79–85 (2009).

197. Saksida, A., Duh, D., Korva, M. & Avsic-Zupanc, T. Dobrava virus RNA load in patients who have hemorrhagic fever with renal syndrome. *J. Infect. Dis.* **197,** 681–5 (2008).

198. Klempa, B. *et al.* First molecular identification of human Dobrava virus infection in Central Europe. *Joural Clin. Microbiol.* **42,** 1322–1325 (2004).

199. Papa, A. *et al.* Isolation of Dobrava virus from *Apodemus flavicollis* in Greece. *J. Clin. Microbiol.* **39,** 2291–2293 (2001).

200. Jakab, F. *et al.* Detection of Dobrava Hantaviruses in *Apodemus agrarius* mice in the Transdanubian region of Hungary. *Virus Res.* **128,** 149–52 (2007).

201. Sironen, T., Vaheri, A. & Plyusnin, A. Phylogenetic evidence for the distinction of Saaremaa and Dobrava Hantaviruses. *Virol. J.* **2,** 1–6 (2005).

202. Lundkvist, Å., Vasilenko, V., Golovljova, I., Plyusnin, A. & Vaheri, A. Human Dobrava Hantavirus infections in Estonia. *Lancet* **352,** 1998 (1998).

203. Nichol, S. T. *et al.* Characteristics of Puumala and Dobrava Infections in Croatia. *J. Med. Virol.* **551,** 542–551 (2002).

204. Nemirov, K. *et al.* Genetic characterization of new Dobrava Hantavirus isolate from Greece. *J. Med. Virol.* **69,** 408–16 (2003).

205. Weidmann, M. *et al.* Identification of genetic evidence for Dobrava virus spillover in rodents by nested reverse transcription (RT)-PCR and TaqMan RT-PCR. *J. Clin. Microbiol.* **43,** 808–812 (2005).

206. Plyusnin, A. *et al.* Dobrava Hantavirus in Russia. *Lancet* **353,** 207 (1999).

207. Sar, N., Hofmann, J., Canpolat, A. T., Türk, A. & Ettinger, J. Dobrava Hantavirus infection complicated by Panhypopituitarism Istanbul, Turkey 2010. *Emerg. Infect. Dis.* **18,** 1180–1183 (2012).

208. Van Thiel, P.-P. A. M. *et al.* Fatal human rabies due to Duvenhage virus from a bat in Kenya: failure of treatment with coma-induction, ketamine, and antiviral drugs. *PLoS Negl. Trop. Dis.* **3,** e428 (2009).

209. Paweska, J. T. *et al.* Fatal human infection with Rabies-related Duvenhage Virus, South Africa. *Emerg. Infect. Dis.* **12,** 1965–1967 (2006).

210. Smith, A., Prato, C. & Skilling, D. Characterization of two new serotypes of San Miguel sea lion virus. *Intervirology* **8,** 30–36 (2008).

211. Lafon, M., Bourhy, H. & Sureau, P. Immunity against the European bat rabies (Duvenhage) virus induced by rabies vaccines: an experimental study in mice. *Vaccine* **6,** 362–368 (1988).

212. Alatoom, A. & Payne, D. An overview of Arboviruses and Bunyaviruses. *Lab. Med.* **40,** 237–240 (2009).

213. Reisen, W. K., Kramer, L. D., Chiles, R. E., Green, E.-G. N. & Martinez, V. M. Encephalitis virus persistence in California birds: preliminary studies with house finches. *J. Med. Entomol.* **38,** 393–399 (2001).

214. Harvala, H. *et al.* Case report: Eastern equine encephalitis virus imported to the UK. *J. Med. Virol.* **81,** 305–308 (2009).

215. Hachiya, M., Osborne, M., Stinson, C. & Werner, B. G. Human eastern equine encephalitis in Massachusetts: predictive indicators from mosquitoes collected at 10 long-term trap sites, 1979-2004. *Am. J. Trop. Med. Hyg.* **76,** 285–92 (2007).

216. Aguilar, P. V *et al.* Endemic eastern equine encephalitis in the Amazon region of Peru. *Am. J. Trop. Med. Hyg.* **76,** 293–8 (2007).

217. Armstrong, P. M. & Andreadis, T. G. Eastern equine encephalitis virus in mosquitoes and their role as bridge vectors. *Emerg. Infect. Dis.* **16,** 1869–1874 (2010).

218. Nolen-Walston, R. *et al.* Eastern equine encephalitis in 9 South American camelids. *J. Vet. Intern. Med.* **21,** 846–52 (2007).

219. Loftin, K. C. *et al.* Five-year surveillance of West Nile and Eastern equine encephalitis viruses in southeastern Virginia. *J. Environ. Health* **68,** 33–4 (2006).

220. Macdonald, J. *et al.* Molecular phylogeny of Edge Hill virus supports its position in the Yellow fever virus group and identifies a new genetic variant. *Evol. Bioinforma.* **6,** 91–96 (2010).

221. Aaskov, J. G., Phillips, D. A. & Wiemers, M. A. Possible clinical infection with Edge Hill virus. *Trans. R. Soc. Trop. Med. Hyg.* **87,** 452–453 (1993).

222. Canelli, E. *et al.* Encephalomyocarditis virus infection in an Italian zoo. *Virol. J.* **7,** 64 (2010).

223. Oberste, M. S. *et al.* Human febrile illness caused by Encephalomyocarditis virus infection, Peru. *Emerg. Infect. Dis.* **15,** 640–6 (2009).

224. Antoniou, M. & Psaroulaki, A. Rats as indicators of the presence and dispersal of pathogens in Cyprus: ectoparasites, parasitic helminths, enteric bacteria, and Encephalomyocarditis virus. *Vector borne zoonotic Dis.* **10,** 867–873 (2010).

225. Billinis, C. Encephalomyocarditis virus infection in wildlife species in Greece. *J. Wildl. Dis.* **45,** 522–526 (2009).

226. Vanderhallen, H. & Koenen, F. Identification of Encephalomyocarditis virus in clinical samples by reverse transcription-PCR followed by genetic typing using sequence analysis. *J. Clin. Microbiol.* **36,** 3463–3467 (1998).

227. Fooks, A. R. *et al.* Case Report : Isolation of a European Bat Lyssavirus Type 2a From a Fatal Human Case of Rabies Encephalitis. *J. Med. Virol.* **71,** 281–289 (2003).

228. Johnson, N., Wakeley, P. R., Brookes, S. M. & Fooks, A. R. European bat lyssavirus type 2 RNA in *Myotis daubentonii*. *Emerg. Infect. Dis.* **12,** 1142–4 (2006).

229. Brookes, S. M. *et al.* European bat lyssavirus in Scottish bats. *Emerg. Infect. Dis.* **11,** 572–8 (2005).

230. Jakava-Viljanen, M., Lilley, T., Kyheröinen, E.-M. & Huovilainen, A. First encounter of European bat lyssavirus type 2 (EBLV-2) in a bat in Finland. *Epidemiol. Infect.* **138,** 1581–5 (2010).

231. Megali, A. *et al.* Surveillance for European bat lyssavirus in Swiss bats. *Arch. Virol.* **155,** 1655–62 (2010).

232. Takumi, K., Lina, P. H. C., Poel, W. H. M. Van Der, Kramps, J. A. & Giessen, J. W. B. Van Der. Public health risk analysis of European bat lyssavirus infection in The Netherlands. *Epidemiol. Infect.* **137,** 803–9 (2009).

233. Dacheux, L. *et al.* European bat lyssavirus transmission among cats, Europe. *Emerg. Infect. Dis.* **15,** 280–284 (2009).

234. Smreczak, M., Oràowska, A. & Zmudzinski, J. F. First case of the European bat lyssavirus type 1B in bats *(Eptesicus serotinus)* in Poland in retrospective study. *Bull. Vet. Inst. Pulawy* **53,** 589–595 (2009).

235. Vázquez-morón, S. *et al.* Endemic circulation of European bat lyssavirus Type 1 in Serotine bats, Spain. *Emerg. Infect. Dis.* **14,** 1263–1266 (2008).

236. Amengual, B., Bourhy, H., López-Roig, M. & Serra-Cobo, J. Temporal dynamics of European bat Lyssavirus type 1 and survival of *Myotis myotis* bats in natural colonies. *PLoS One* **2,** e566 (2007).

237. Tjørnehøj, K., Fooks, A. R., Agerholm, J. S. & Rønsholt, L. Natural and experimental infection of sheep with European bat lyssavirus type-1 of Danish bat origin. *J. Comp. Pathol.* **134,** 190–201 (2006).

238. Müller, T. *et al.* Spill-over of European bat lyssavirus type 1 into a Stone Marten *(Martes foina)* in Germany. *J. Vet. Med.* **51,** 49–54 (2004).

239. Serra-Cobo, J., Amengual, B., Abellán, C. & Bourhy, H. European bat lyssavirus infection in Spanish bat populations. *Emerg. Infect. Dis.* **8,** 413–20 (2002).

240. Wellenberg, G. J. *et al.* Presence of European bat lyssavirus RNAs in apparently healthy *Rousettus aegyptiacus* bats. *Arch. Virol.* **147,** 349–61 (2002).

241. Banyard, A. C. *et al.* Repeated detection of European bat lyssavirus type 2 in dead bats found at a single roost site in the UK. *Arch. Virol.* **154,** 1847–50 (2009).

242. Harris, S. L. *et al.* European bat lyssaviruses: distribution, prevalence and implications for conservation. *Biol. Conserv.* **131,** 193–210 (2006).

243. Coffey, L. L. *et al.* Serologic evidence of widespread Everglades virus activity in dogs, Florida. *Emerg. Infect. Dis.* **12,** 1873–9 (2006).

244. Coffey, L. L. & Weaver, S. C. Susceptibility of *Ochlerotatus taeniorhynchus* and *Culex nigripalpus* for Everglades virus. *Am. J. Trop. Med. Hyg.* **73,** 11–16 (2005).

245. Calisher, C. H. *et al.* Everglades virus infection in man. *South. Med. J.* **73,** 1548 (1980).

246. Brown, C. M. & Timoney, P. J. Getah virus infection of Indian horses. *Trop. Anim. Health Prod.* **30,** 241–52 (1998).

247. Cross, H., Province, N. E. & Ecija, N. Isolation of Getah virus from Nueva Ecija Province, Republic of the Philippines. *Trans. R. Soc. Trop. Med. Hyg.* **75,** 312–313 (1981).

248. Sugiyama, I. *et al.* Serological survey of arthropod-borne viruses among wild boars in Japan. *J. Vet. Med. Sci.* **71,** 1059–61 (2009).

249. Sun, X. *et al.* Distribution of arboviruses and mosquitoes in northwestern Yunnan Province, China. *Vector borne zoonotic Dis.* **9,** 623–630 (2009).

250. Wekesa, S. N., Inoshima, Y., Murakami, K. & Sentsui, H. Genomic analysis of some Japanese isolates of Getah virus. *Vet. Microbiol.* **83,** 137–46 (2001).

251. Wen, J.-S. *et al.* Genomic analysis of a Chinese isolate of Getah-like virus and its phylogenetic relationship with other Alphaviruses. *Virus Genes* **35,** 597–603 (2007).

252. Weaver, S. C. *et al.* Guanarito virus (Arenaviridae) isolates from endemic and outlying localities in Venezuela: sequence comparisons among and within strains isolated from Venezuelan hemorrhagic fever patients and rodents. *Virology* **266,** 189–95 (2000).

253. Milazzo, M. L. *et al.* Diversity among Tacaribe serocomplex viruses (family Arenaviridae) naturally associated with the white-throated woodrat *(Neotoma albigula)* in the southwestern United States. *Vector borne zoonotic Dis.* **8,** 523–40 (2008).

254. Weaver, S. C. *et al.* Extreme genetic diversity among Pirital virus (Arenaviridae) isolates from western Venezuela. *Virology* **285,** 110–8 (2001).

255. Tesh, R., Jahrling, P., Salas, R. & Shope, R. Description of Guanarito virus (Arenaviridae: Arenavirus), the etiologic agent of Venezuelan hemorrhagic fever. *Am. J. Trop. Med. Hyg.* **50,** 452–459 (1994).

256. Gonzalez, J. P., Emonet, S., Lamballerie, X. & Charrel, R. Arenaviruses: wildlife and emerging zoonotic diseases: the biology, circumstances and consequences of cross-species transmission. *Curr. Top. Microbiol. Immunol.* **315,** 253–288 (2007).

257. Fulhorst, C. *et al.* Genetic diversity between and within the Arenavirus species indigenous to western Venezuela. *Virology* **378,** 205–213 (2008).

258. Charrel, R. & Lamballerie, X. Arenaviruses other than Lassa virus. *Antiviral Res.* **57,** 89–100 (2003).

259. Aguilar, P. V *et al.* Guaroa virus infection among humans in Bolivia and Peru. *Am. J. Trop. Med. Hyg.* **83,** 714–21 (2010).

260. Song, K.-J. *et al.* Muju virus, a novel Hantavirus harboured by the arvicolid rodent *Myodes regulus* in Korea. *J. Gen. Virol.* **88,** 3121–9 (2007).

261. Yan, L. *et al.* Landscape elements and Hantaan virus – related hemorrhagic fever with renal syndrome, People’s Republic of China. *Emerg. Infect. Dis.* **13,** 1301–1306 (2007).

262. Mathes, R. W., Page, W. F., Crawford, H. M., McBean, a M. & Miller, R. N. Long-term sequelae of hemorrhagic fever with renal syndrome attributable to Hantaan virus in Korean War veterans. *Mil. Med.* **170,** 315–9 (2005).

263. Sames, W. J. *et al.* Ecology of Hantaan virus at Twin Bridges Training Area, Gyeonggi Province, Republic of Korea, 2005–2007. *J. Vector Ecol.* **34,** 225–231 (2009).

264. Zou, Y. *et al.* Molecular diversity and phylogeny of Hantaan virus in Guizhou, China: evidence for Guizhou as a radiation center of the present Hantaan virus. *J. Gen. Virol.* **89,** 1987–97 (2008).

265. Zhang, Y. *et al.* Hantavirus outbreak associated with laboratory rats in Yunnan, China. *Infect. Genet. Evol.* **10,** 638–44 (2010).

266. Kao, C. L., Chen, C. J., Yen, T. S., Lien, J. C. & Yang, C. S. Seroepidemiology of Hantaan virus infection in Taiwan. *J. Med. Virol.* **50,** 309–13 (1996).

267. Lee, H. W., Lee, P. W. & Johnson, K. M. Isolation of the etiologic agent of Korean hemorrhagic fever, 1978. *J. Infect. Dis.* **190,** 1711–21 (2004).

268. Song, J. W. *et al.* Genetic diversity of *Apodemus agrarius*-borne Hantaan virus in Korea. *Virus Genes* **21,** 227–32 (2000).

269. Chua, K. B. *et al.* Tioman virus, a novel Paramyxovirus isolated from fruit bats in Malaysia. *Virology* **283,** 215–29 (2001).

270. Playford, E. G. *et al.* Human Hendra virus encephalitis associated with equine outbreak, Australia, 2008. *Emerg. Infect. Dis.* **16,** 219–223 (2010).

271. Field, H. Hendra virus outbreak with novel clinical features, Australia. *Emerg. Infect. Dis.* **16,** 2008–2010 (2010).

272. Field, H. Hendra virus infection risks. *Curr. Top. Microbiol. Immunol.* **14,** 77 – 78 (2009).

273. Wong, K. T. *et al.* Human Hendra virus infection causes acute and relapsing encephalitis. *Neuropathol. Appl. Neurobiol.* **35,** 296–305 (2009).

274. Plowright, R. K. *et al.* Reproduction and nutritional stress are risk factors for Hendra virus infection in Little red flying foxes *(Pteropus scapulatus)*. *Proc. Biol. Sci.* **275,** 861–9 (2008).

275. Field, H. E. *et al.* Epidemiological perspectives on Hendra virus infection in horses and flying foxes. *Aust. Vet. J.* **85,** 268–70 (2007).

276. Barker, S. The Australian paralysis tick may be the missing link in the transmission of Hendra virus from bats to horses to humans. *Med. Hypotheses* **60,** 481–483 (2003).

277. Hayman, D. T. S. *et al.* Evidence of Henipavirus infection in West African fruit bats. *PLoS One* **3,** e2739 (2008).

278. Breed, A. C., Field, H. E., Smith, C. S., Edmonston, J. & Meers, J. Bats without borders: long-distance movements and implications for disease risk management. *Ecohealth* **7,** 204–12 (2010).

279. Chong, H. T., Abdullah, S. & Tan, C. T. Nipah virus and bats. *Neurol. Asia* **14,** 73–76 (2009).

280. Pavio, N., Meng, X.-J. & Renou, C. Zoonotic Hepatitis E: animal reservoirs and emerging risks. *Vet. Res.* **41,** 46 (2010).

281. Kaba, M., Davoust, B., Marié, J.-L. & Colson, P. Detection of Hepatitis E virus in wild boar *(Sus scrofa)* livers. *Vet. J.* **186,** 259–61 (2010).

282. Kaba, M. *et al.* Hepatitis E virus infection in sheltered homeless persons, France. *Emerg. Infect. Dis.* **16,** 1761–1764 (2010).

283. Meng, X. J. Hepatitis E virus: animal reservoirs and zoonotic risk. *Vet. Microbiol.* **140,** 256–65 (2010).

284. Reuter, G., Fodor, D., Forgách, P., Kátai, A. & Szucs, G. Characterization and zoonotic potential of endemic Hepatitis E virus (HEV) strains in humans and animals in Hungary. *J. Clin. Virol.* **44,** 277–81 (2009).

285. Takahashi, K., Kitajima, N., Abe, N. & Mishiro, S. Complete or near-complete nucleotide sequences of hepatitis E virus genome recovered from a wild boar, a deer, and four patients who ate the deer. *Virology* **330,** 501–5 (2004).

286. Chang, Y. *et al.* Zoonotic risk of Hepatitis E virus (HEV): A study of HEV infection in animals and humans in suburbs of Beijing. *Hepatol. Res.* **39,** 1153–8 (2009).

287. Worm, H. C., van der Poel, W. H. M. & Brandstätter, G. Hepatitis E: an overview. *Microbes Infect.* **4,** 657–66 (2002).

288. Elmore, D. & Eberle, R. Monkey B virus (Cercopithecine Herpesvirus 1). *Comp. Med.* **58,** 11–21 (2008).

289. Ritz, N., Curtis, N., Buttery, J. & Babl, F. E. Monkey bites in travelers: should we think of Herpes B virus? *Pediatr. Emerg. Care* **25,** 529–31 (2009).

290. Huff, J. L. & Berry, P. A. B-virus (Cercopithecine Herpesvirus 1) infection in humans and macaques: potential for zoonotic disease. *Emerg. Infect. Dis.* **9,** 246–250 (2003).

291. Jainkittivong, A. & Langlais, R. P. Herpes B virus infection. *Oral Surg. Oral Med. Oral Pathol. Oral Radiol. Endod.* **85,** 399–403 (1998).

292. Espana, C. Herpesvirus simiae infection in *Macaca radiata*. *Am. J. Phys. Anthr.* **38,** 447–454 (2005).

293. Jensen, K., Alvarado-ramy, F., González-martínez, J., Kraiselburd, E. & Rullán, J. B-virus and free-ranging macaques Puerto Rico. *Emerg. Infect. Dis.* **10,** 494–496 (2004).

294. Lee, F., Lin, Y.-J., Deng, M.-C., Lee, T.-Y. & Huang, C.-C. Prevalence of antibody reaction with Cercopithecine Herpesvirus 1 antigen in *Macaca cyclopis Macaca fascicularis*, and *Papio anubis* in Taiwan. *J. Med. Primatol.* **36,** 343–7 (2007).

295. Wilson, R. B., Holscher, M. a., Chang, T. & Hodges, J. R. Fatal Herpesvirus Simiae (B Virus) infection in a Patas monkey *(Erythrocebus Patas)*. *J. Vet. Diagnostic Investig.* **2,** 242–244 (1990).

296. Andrade, M. R. *et al.* Prevalence of antibodies to selected viruses in a long-term closed breeding colony of Rhesus macaques *(Macaca mulatta)* in Brazil. *Am. J. Primatol.* **59,** 123–8 (2003).

297. Coulibaly, C. *et al.* A natural asymptomatic herpes B virus infection in a colony of laboratory brown capuchin monkeys *(Cebus apella)*. *Lab. Anim.* **38,** 432–438 (2004).

298. Engel, G. & Jones-Engel, L. Human exposure to Herpesvirus B–seropositive macaques, Bali, Indonesia. *Emerg. Infect. Dis.* **8,** 789–795 (2002).

299. Jones-Engel, L. *et al.* Temple monkeys and health implications of commensalism, Kathmandu, Nepal. *Emerg. Infect. Dis.* **12,** 900–906 (2006).

300. Johnson, B. W. *et al.* Ilheus virus isolate from a human, Ecuador. *Emerg. Infect. Dis.* **13,** 956–8 (2007).

301. Anderson, C. R., Trinidad, T., Aitken, T. H. G., Downs, W. G. & Indies, W. The isolation of Ilheus virus from wild caught forest mosquitoes in Trinidad. *Unknon...need to find* 621–625 (1947).

302. Li, Y. *et al.* Persistent circulation of highly pathogenic influenza H5N1virus in Lake Qinghai Area of China. *Avian Dis.* **54,** 821–829 (2010).

303. Desvaux, S. *et al.* Highly pathogenic Avian influenza virus (H5N1) outbreak in captive wild birds and cats, Cambodia. *Emerg. Infect. Dis.* **15,** 475–479 (2009).

304. Roberton, S. I. *et al.* Avian influenza H5N1 in viverrids: implications for wildlife health and conservation. *Proc. Biol. Sci.* **273,** 1729–32 (2006).

305. Yee, K. S., Carpenter, T. E. & Cardona, C. J. Epidemiology of H5N1 Avian influenza. *Comp. Immunol. Microbiol. Infect. Dis.* **32,** 325–40 (2009).

306. Kayali, G., Ortiz, E. J., Chorazy, M. L. & Gray, G. C. Evidence of previous avian influenza infection among US turkey workers. *Zoonoses Public Health* **57,** 265–72 (2010).

307. Keawcharoen, J. *et al.* Avian influenza H5N1 in tigers and leopards. *Emerg. Infect. Dis.* **10,** 2189–91 (2004).

308. Stoops, A. C. *et al.* H5N1 surveillance in migratory birds in Java, Indonesia. *Vector borne zoonotic Dis.* **9,** 695–702 (2009).

309. Yamamoto, Y., Nakamura, K., Yamada, M. & Ito, T. Zoonotic risk for influenza A (H5N1) infection in wild swan feathers. *J. Vet. Med. Sci.* **71,** 1549–51 (2009).

310. Blanc, A. *et al.* Serologic evidence of influenza A and B viruses in South American fur seals *(Arctocephalus australis)*. *J. Wildl. Dis.* **45,** 519–21 (2009).

311. Osterhaus, a. D., Rimmelzwaan, G. F., Martina, B. E. E., Bestebroer, T. M. & Fouchier, R. A. M. Influenza B virus in seals. *Science* **288,** 1051–1053 (2000).

312. Putkuri, N., Vaheri, A. & Vapalahti, O. Prevalence and protein specificity of human antibodies to Inkoo virus infection. *Clin. Vaccine Immunol.* **14,** 1555–62 (2007).

313. Mayo, D. *et al.* Jamestown Canyon Virus: seroprevalence in Connecticut. *Emerg. Infect. Dis.* **7,** 8–9 (2001).

314. Murdock, C. C., Olival, K. J. & Perkins, S. L. Molecular identification of host feeding patterns of snow-melt mosquitoes (Diptera: Culicidae): potential implications for the transmission ecology of Jamestown Canyon virus. *J. Med. Entomol.* **47,** 226–229 (2010).

315. Zheng, H. *et al.* Jamestown Canyon virus detection in human tissue specimens. *J. Clin. Pathol.* **60,** 787–93 (2007).

316. Nelson, D. M. *et al.* Prevalence of antibodies against Saint Louis encephalitis and Jamestown Canyon viruses in California horses. *Comp. Immunol. Microbiol. Infect. Dis.* **27,** 209–15 (2004).

317. Sahu, S. P. *et al.* Isolation of Jamestown Canyon virus (California virus group) from vesicular lesions of a horse. *J. Vet. Diagnostic Investig.* **12,** 80–83 (2000).

318. Andreadis, T. G., Anderson, J. F., Armstrong, P. M. & Main, A. J. Isolations of Jamestown canyon virus (Bunyaviridae: Orthobunyavirus) from field collected mosquitoes (Diptera: culicidaw) in connectivcut USA: A ten-year analysis. *Vector-borne zoonotic Dis.* **8,** 175–188 (2008).

319. Armstrong, P. M. & Andreadis, T. G. Genetic relationships of Jamestown Canyon virus strains infecting mosquitoes collected in Connecticut. *Am. J. Trop. Med. Hyg.* **77,** 1157–62 (2007).

320. Grimstad, P. R., Schmitt, S. M. & Williams, D. G. Prevalence of neutralizing antibody to Jamestown Canyon virus (California group) in populations of elk and moose in northern Michigan and Ontario, Canada. *J. Wildl. Dis.* **22,** 453–458 (1986).

321. Grieve, S. Japanese encephalitis. *Pract. Nurs.* **21,** (2010).

322. Nga, P. T. Shift in Japanese encephalitis virus (JEV) genotype circulating in northern Vietnam: implications for frequent introductions of JEV from Southeast Asia to East Asia. *J. Gen. Virol.* **85,** 1625–1631 (2004).

323. Nitatpattana, N. *et al.* Change in Japanese encephalitis virus distribution, Thailand. *Emerg. Infect. Dis.* **14,** 1762–5 (2008).

324. Erlanger, T. E., Weiss, S., Keiser, J., Utzinger, J. & Wiedenmayer, K. Past, present, and future of Japanese encephalitis. *Emerg. Infect. Dis.* **15,** (2009).

325. Singha, H. *et al.* Complete genome sequence analysis of Japanese encephalitis virus isolated from a horse in India. *Arch. Virol.* **158,** 113–22 (2013).

326. Lindahl, J. F. *et al.* Circulation of Japanese encephalitis virus in pigs and mosquito vectors within Can Tho city, Vietnam. *PLoS Negl. Trop. Dis.* **7,** (2013).

327. Charrel, R. N. & de Lamballerie, X. Zoonotic aspects of Arenavirus infections. *Vet. Microbiol.* **140,** 213–20 (2010).

328. Weaver, S. C. *et al.* Extreme genetic diversity among Pirital virus (Arenaviridae) isolates from western Venezuela. *Virology* **285,** 110–8 (2001).

329. Cajimat, M. N. B. *et al.* Diversity among Tacaribe serocomplex viruses (family Arenaviridae) naturally associated with the Mexican woodrat *(Neotoma mexicana)*. *Virus Res.* **133,** 211–7 (2008).

330. Arata, A. A. & Gratz, N. G. The structure of rodent faunas associated with arenaviral infections. *Bull. World Health Organ.* **52,** 621 (1975).

331. Enria, D., Briggiler, A. & Feuillade, M. An overview of the epidemiological, ecological and preventive hallmarks of Argentine haemorrhagic fever (Junin virus). *Bull. Inst. Pasteur* **96,** 103–114 (1998).

332. Mills, J., Ellis, B. & Jr, K. M. Junin virus activity in rodents from endemic and nonendemic loci in central Argentina. *Am. J. Trop. Med. Hyg.* **44,** 589–597 (1991).

333. Polop, J. *et al.* Spatial variation in abundance of the junin virus hosts in endemic and nonendemic Argentine haemorrhagic fever zones. *Austral Ecol.* **32,** 245–253 (2007).

334. Chiappero, M. B., Gardenal, C. N. & Panzetta-Dutari, G. M. Isolation and characterization of microsatellite markers in *Calomys musculinus* (Muridae, Sigmodontinae, Phyllotini), the natural reservoir of Junin virus. *Mol. Ecol. Notes* **5,** 593–595 (2005).

335. García, J. B. *et al.* Genetic diversity of the Junin virus in Argentina: geographic and temporal patterns. *Virology* **272,** 127–36 (2000).

336. Polop, F. *et al.* On the relationship between the environmental history and the epidemiological situation of Argentine hemorrhagic fever. *Ecol. Res.* **23,** 217–225 (2008).

337. Oliveira, R. C. *et al.* Genetic characterization of a Juquitiba-like viral lineage in *Oligoryzomys nigripes* in Rio de Janeiro, Brazil. *Acta Trop.* **112,** 212–8 (2009).

338. Delfraro, A. *et al.* Juquitiba-like Hantavirus from 2 nonrelated rodent species, Uruguay. *Emerg. Infect. Dis.* **14,** 1447–1451 (2008).

339. Calisher, C. H., Oro, J. B., Lord, R. D., Sabattini, M. S. & Karabatsos, N. Kairi virus identified from a fabrile horse in Argentina. *Am. J. Trop. Med. Hyg.* **39,** 519–521 (1988).

340. Soto, V. *et al.* Complete nucleotide sequences of the small and medium RNA genome segments of Kairi virus (family Bunyaviridae). *Arch. Virol.* **154,** 1555–8 (2009).

341. Mehla, R. *et al.* Recent ancestry of Kyasanur Forest disease virus. *Emerg. Infect. Dis.* **15,** 1431–7 (2009).

342. Wang, J. Isolation of Kyasanur Forest disease virus from febrile patient, Yunnan, China. *Emerg. Infect. Dis.* **15,** 326–328 (2009).

343. Pattnaik, P. Kyasanur Forest disease: an epidemiological view in India. *Rev. Med. Virol.* **16,** 151–65 (2006).

344. Armstrong, P. M. & Andreadis, T. G. A new genetic variant of La Crosse virus (Bunyaviridae) isolated from New England. *Am. J. Trop. Med. Hyg.* **75,** 491–6 (2006).

345. Borucki, M. K., Kempf, B. J., Blitvich, B. J., Blair, C. D. & Beaty, B. J. La Crosse virus: replication in vertebrate and invertebrate hosts. *Microbes Infect.* **4,** 341–50 (2002).

346. Sokol, D. K., Kleiman, M. B. & Garg, B. P. La Crosse viral encephalitis mimics herpes simplex viral encephalitis. *Pediatr. Neurol.* **25,** 413–5 (2001).

347. Rust, R. S., Thompson, W. H., Matthews, C. G., Beaty, B. J. & Chun, R. W. M. Topical review: La Crosse and other forms of California encephalitis. *J. Child Neurol.* **14,** 1–14 (1999).

348. Balfour, H. H., Edelman, C. K., Bauer, H. & Siem, R. A. California arbovirus (La Crosse) infections. III. Epidemiology of California encephalitis in Minnesota. *J. Infect. Dis.* **133,** 293–301 (1976).

349. Gerhardt, R. R. *et al.* First isolation of La Crosse virus from naturally infected *Aedes albopictus*. *Emerg. Infect. Dis.* **7,** 807–811 (2001).

350. Haddow, A. D. & Odoi, A. The incidence risk, clustering, and clinical presentation of La Crosse virus infections in the eastern United States, 2003-2007. *PLoS One* **4,** e6145 (2009).

351. Lambert, A. J. *et al.* La Crosse virus in *Aedes albopictus* mosquitoes, Texas, USA, 2009. *Emerg. Infect. Dis.* **16,** 856–8 (2010).

352. Polop, F. J. *et al.* Temporal and spatial host abundance and prevalence of Andes Hantavirus in southern Argentina. *Ecohealth* **7,** 176–84 (2010).

353. Carroll, D. S. *et al.* Hantavirus pulmonary syndrome in Central Bolivia: relationships between reservoir hosts, habitats, and viral genotypes. *Am. J. Trop. Med. Hyg.* **72,** 42–6 (2005).

354. Levis, S. *et al.* Hantavirus pulmonary syndrome in northwestern Argentina: circulation of Laguna Negra virus associated with *Calomys callosus*. *Am. J. Trop. Med. Hyg.* **71,** 658–63 (2004).

355. Yahnke, C. J., Meserve, P. L., Ksiazek, T. G. & Mills, J. N. Patterns of infection with Laguna Negra virus in wild populations of *Calomys laucha* in the central Paraguayan chaco. *Am. J. Trop. Med. Hyg.* **65,** 768–76 (2001).

356. Raboni, S. M. *et al.* Evidence of circulation of Laguna Negra-like Hantavirus in the central west of Brazil: case report. *J. Clin. Virol.* **45,** 153–6 (2009).

357. Ferrer, J. F. *et al.* Hantavirus infection in people inhabiting a highly endemic region of the Gran Chaco territory, Paraguay: association with *Trypanosoma cruzi* infection, epidemiological features and haematological characteristics. *Ann. Trop. Med. Parasitol.* **97,** 269–80 (2003).

358. Padula, P. J. *et al.* Genetic diversity, distribution, and serological features of Hantavirus infection in five countries in South America. *J. Clin. Microbiol.* **38,** 3029–35 (2000).

359. Cross, H., Province, N. E. & Ecija, N. Isolation of Getah virus from Nueva Ecija Province, Republic of the Philippines. *Trans. R. Soc. Trop. Med. Hyg.* **75,** 312–313 (1981).

360. Safronetz, D. *et al.* Detection of Lassa virus, Mali. *Emerg. Infect. Dis.* **16,** 1123–1127 (2010).

361. Emmerich, P., Günther, S. & Schmitz, H. Strain-specific antibody response to Lassa virus in the local population of west Africa. *J. Clin. Virol.* **42,** 40–44 (2008).

362. Mcconnell, R. *et al.* Imported Lassa Fever, Pennsylvania, USA, 2010. *Emerg. Infect. Dis.* **16,** 1598–1601 (2010).

363. Cooke, F. J. & Shapiro, D. S. ProMED update - Lassa fever case imported to Germany from Sierra Leone. *Int. J. Infect. Dis.* **10,** 341–342 (2006).

364. Macher, A. M. & Wolfe, M. S. Historical Lassa Fever reports and 30-year clinical update. *Emerg. Infect. Dis.* **12,** 835–837 (2006).

365. Omilabu, S. A. *et al.* Lassa fever, Nigeria, 2003 and 2004. *Emerg. Infect. Dis.* **11,** 1642–4 (2005).

366. Chakarala, A. Imported Lassa Fever-New Jersey, 2004. *J. Am. Med. Assoc.* **292,** 2828–2830 (2004).

367. Crowcroft, N. S. *et al.* The public health response to a case of Lassa fever in London in 2000. *J. Infect.* **48,** 221–8 (2004).

368. Meulen, J. Ter *et al.* Short Communication: Lassa fever in Sierra Leone UN peacekeepers are at risk. *Trop. Med. Int. Heal.* **6,** 83–84 (2001).

369. Garcia, F. L. B. *et al.* A retrospective laboratory analysis of clinically diagnosed Lassa Fever cases in a tertiary hospital in Nigeria. *Int. J. Infect. Dis.* **14,** e209–e209 (2010).

370. Okogbenin, S. A. *et al.* Prevalence and presentation of Lassa fever in Nigerian children. *Int. J. Infect. Dis.* **14,** e380–e380 (2010).

371. Ehichioya, D. U. *et al.* Lassa Fever, Nigeria, 2005-2008. *Emerg. Infect. Dis.* **16,** 1040–1042 (2010).

372. Kernéis, S. *et al.* Prevalence and risk factors of Lassa seropositivity in inhabitants of the forest region of Guinea: a cross-sectional study. *PLoS Negl. Trop. Dis.* **3,** e548 (2009).

373. Ehichioya1, D. U., Omilabu, S., Asogun, D. A., Hass, M. & Gunther, S. Clinical and serological evidence of Lassa Fever in Edo State, Nigeria. *Int. J. Infect. Dis.* **12,** e331–e332 (2008).

374. Fair, J. *et al.* Lassa virus-infected rodents in refugee camps in Guinea: a looming threat to public health in a politically unstable region. *Vector Borne Zoonotic Dis.* **7,** 167–71 (2007).

375. Lecompte, E. *et al.* *Mastomys natalensis* and Lassa fever, West Africa. *Emerg. Infect. Dis.* **12,** 1971–4 (2006).

376. Niklasson, B. *et al.* A new Picornavirus isolated from bank voles *(Clethrionomys glareolus)*. *Virology* **255,** 86–93 (1999).

377. Niklasson, B., Almqvist, P. R., Hörnfeldt, B. & Klitz, W. Sudden infant death syndrome and Ljungan virus. *Forensic Sci. Med. Pathol.* **5,** 274–9 (2009).

378. Hauffe, H. C. *et al.* Ljungan virus detected in bank voles *(Myodes glareolus)* and yellow-necked mice *(Apodemus flavicollis)* from Northern Italy. *J. Wildl. Dis.* **46,** 262–6 (2010).

379. Donoso Mantke, O., Kallies, R., Niklasson, B., Nitsche, A. & Niedrig, M. A new quantitative real-time reverse transcriptase PCR assay and melting curve analysis for detection and genotyping of Ljungan virus strains. *J. Virol. Methods* **141,** 71–7 (2007).

380. Briese, T. *et al.* Genetic detection and characterization of Lujo virus, a new hemorrhagic fever-associated arenavirus from southern Africa. *PLoS Pathog.* **5,** e1000455 (2009).

381. Paweska, J. T. *et al.* Nosocomial outbreak of novel arenavirus infection, southern Africa. *Emerg. Infect. Dis.* **15,** 1598–602 (2009).

382. Rousseau, M., Saron, M., Brouqui, P. & Bourgeade, A. Lymphocytic choriomeningitis virus in southern France: four case reports and a review of the literature. *Eur. J. Epidemiol.* **13,** 817–823 (1997).

383. Karamany, R. El & Imam, I. Antibodies to Lymphocytic choriomeningitis virus in wild rodent sera in Egypt. *J. Hyg. Epidemiol. Microbiol. Immunol.* **35,** 97–103 (1991).

384. Morita, C., Matsuura, Y. & Fujii, H. Isolation of Lymphocytic choriomeningitis virus from wild house mice *(Mus musculus)* in Osaka Port, Japan. *J. Vet. Med. Sci.* **53,** 889–892 (1991).

385. Becker, S., Bennett, M., Stewart, J. & Hurst, J. Serological survey of virus infection among wild house mice *(Mus domesticus)* in the UK. *Lab. Anim.* **41,** 229–238 (2007).

386. Ledesma, J. *et al.* Independent lineage of Lymphocytic choriomeningitis virus in wood mice *(Apodemus sylvaticus)*, Spain. *Emerg. Infect. Dis.* **15,** 1677–1680 (2009).

387. Tagliapietra, V. *et al.* Spatial and temporal dynamics of Lymphocytic choriomeningitis virus in wild rodents, northern Italy. *Emerg. Infect. Dis.* **15,** 1019–1025 (2009).

388. Marrie, T. & Saron, M. Seroprevalence of Lymphocytic choriomeningitis virus in Nova Scotia. *Am. J. Trop. Med. Hyg.* **58,** 47–49 (1998).

389. Sottosanti, J. *et al.* Serological study of the Lymphocytic choriomeningitis virus (LCMV) in an inner city of Argentina. *J. Med. Virol.* **76,** 285–289 (2005).

390. Asnis, D. S., Muana, O., Kim, D. G., Garcia, M. & Rollin, P. E. Lymphocytic choriomeningitis virus meningitis, New York, NY, USA, 2009. *Emerg. Infect. Dis.* **16,** 328–331 (2010).

391. Asper, M. *et al.* First outbreak of Callitrichid Hepatitis in Germany : genetic characterization of the causative Lymphocytic choriomeningitis virus strains. *Virology* **284,** 203–213 (2001).

392. Ceianu, C. *et al.* Letter to the Editor - Lymphocytic choriomeningitis in a pet store worker in Romania. *Clinial Vaccine Immunol.* **15,** 1749 (2008).

393. Johnson, K. Epidemiology of Machupo virus infection. III. Significance of virological observations in man and animals. *Am. J. Trop. Med. Hyg.* **14,** 816–8 (1965).

394. Murphy, F. Arenavirus taxonomy: a review. *Bull. World Health Organ.* **52,** 387–391 (1975).

395. Report, W. Ebola haemorrhagic fever in Sudan , 1976. *Bull. World Health Organ.* **56,** (1978).

396. Pourrut, X. *et al.* Large serological survey showing cocirculation of Ebola and Marburg viruses in Gabonese bat populations, and a high seroprevalence of both viruses in Rousettus aegyptiacus. *BMC Infect. Dis.* **9,** 159 (2009).

397. Lahm, S., Kombila, M., Swanepoel, R. & Barnes, R. F. W. Morbidity and mortality of wild animals in relation to outbreaks of Ebola haemorrhagic fever in Gabon, 1994-2003. *Trans. R. Soc. Trop. Med. Hyg.* **101,** 64–78 (2007).

398. Towner, J. S. *et al.* Isolation of genetically diverse Marburg viruses from Egyptian fruit bats. *PLoS Pathog.* **5,** e1000536 (2009).

399. Timen, A. *et al.* Response to imported case of Marburg Hemorrhagic Fever, the Netherlands. *Emerg. Infect. Dis.* **15,** 1171–1175 (2009).

400. Swanepoel, R. *et al.* Studies of reservoir hosts for Marburg virus. *Emerg. Infect. Dis.* **13,** 1847–51 (2007).

401. Bausch, D. G. *et al.* Marburg hemorrhagic fever associated with multiple genetic lineages of virus. *N. Engl. J. Med.* **355,** 909–19 (2006).

402. Borchert, M. *et al.* Serosurvey on household contacts of Marburg hemorrhagic fever patients. *Emerg. Infect. Dis.* **12,** 433–439 (2006).

403. Borchert, M. *et al.* Short communication: a cluster of Marburg virus disease involving an infant. *Trop. Med. Int. Heal.* **7,** 902–6 (2002).

404. Gear, J. *et al.* Outbreake of Marburg virus disease in Johannesburg. *Br. Med. J.* **4,** 489–493 (1975).

405. Formenty, P. *et al.* Ebola virus outbreak among wild chimpanzees living in a rain forest of Cote d’Ivoire. *J. Infect. Dis.* **179,** S120–S126 (1999).

406. Martini, G. & Siegert, R. *Marburg virus disease.* *Marbg. virus Dis.* vii+230 (Berlin, Heidelberg, New York: Springer-Verlag., 1971).

407. Smith, D. *et al.* Marburg-virus disease in Kenya. *Lancet* **319,** 816–820 (1982).

408. Slenczka, W. & Klenk, H. The Marburg virus outbreak of 1967 and subsequent episodes. *Marbg. Ebola viruses.* (1999).

409. Gonzalez, J., Nakoune, E., Slenczka, W., Vidal, P. & Morvan, J. Ebola and Marburg virus antibody prevalence in selected populations of the Central African Republic. *Microbes Infect.* **2,** 39–44 (2000).

410. Towner, J. *et al.* Marburgvirus genomics and association with a large hemorrhagic fever outbreak in Angola. *J. Virol.* **80,** 6497 (2006).

411. Tomori, O., Fabiyi, A., Akanni, S., Ademola, S. & McCormick, J. Viral hemorrhagic fever antibodies in Nigerian populations. *Am. J. Trop. Med. Hyg.* **38,** 407–410 (1988).

412. Hassing, R. *et al.* Imported Mayaro virus infection in the Netherlands. *J. Infect.* **61,** 343–345 (2010).

413. JR, T. *et al.* Family Cluster of Mayaro Fever, Venezuela. *Emerg. Infect. Dis.* **10,** 1304–1306 (2004).

414. Thoisy, B. de, Gardon, J., Salas, R. A., Morvan, J. & Kazanji, M. Mayaro virus in wild mammals, French Guiana. *Emerg. Infect. Dis.* **9,** 1326–1329 (2003).

415. Azevedo, R. S. S. *et al.* Mayaro fever virus Brazilian amazon.pdf. *Emerg. Infect. Dis.* **15,** 1830–1832 (2009).

416. Coimbra, T. L. M. *et al.* Mayaro virus: imported cases of human infection in São Paulo State, Brazil. *Rev. Inst. Med. Trop. Sao Paulo* **49,** 221–4 (2007).

417. Receveur, M. C., Grandadam, M., Pistone, T. & Malvy, D. Infection with Mayaro virus in a French traveller returning from the Amazon region, Brazil, January, 2010. *Euro Surveill.* **15,** 2–5 (2010).

418. Tesh, R. B. *et al.* Mayaro virus disease: an emerging mosquito-borne zoonosis in tropical South America. *Clin. Infect. Dis.* **28,** 67–73 (1999).

419. Sabeta, C. *et al.* Mokola virus involved in a human contact (South Africa). *FEMS Immunol. Med. Microbiol.* **58,** 85–90 (2010).

420. Nottidge, H. O., Omobowale, T. O. & Oladiran, O. O. Mokola virus antibodies in humans, dogs, cats, cattle, sheep and goats in Nigeria. *Int. J. Int. J. Appl. Res. Vet. Med.* **5,** 105–106 (2007).

421. Sabeta, C. T. *et al.* Mokola virus in South Africa. *Emerg. Infect. Dis.* **13,** 1371–1374 (2007).

422. Nel, L., Jacobs, J., Jaftha, J., Teichman, B. V. O. N. & Bingham, J. New cases of Mokola virus infection in South Africa: A genotypic comparison of Southern African virus isolates. *Am. J. Trop. Med. Hyg.* **20,** 103–106 (2000).

423. Stephenson, J. Emerging Monkeypox. *J. Am. Med. Assoc.* **304,** 1659 (2010).

424. Sejvar, J. J. *et al.* Human Monkeypox infection: a family cluster in the midwestern United States. *J. Infect. Dis.* **190,** 1833–40 (2004).

425. Mmwr, F. T. H. E. Update: multistate outbreak of monkeypox--Illinois, Indiana, Kansas, Missouri, Ohio, and Wisconsin, 2003. *Arch. Dermatol.* **139,** 1229–1230 (2003).

426. Hutin, Y. J. F. *et al.* Outbreak of human Monkeypox, Democratic Republic of Congo, 1996-1997. *Emerg. Infect. Dis.* **7,** 434–438 (2001).

427. Formenty, P. *et al.* Human Monkeypox Outbreak Caused by Novel Virus Belonging to Congo Basin Clade, Sudan, 2005. *Emerg. Infect. Dis.* **16,** 1539–1546 (2010).

428. Reynolds, M. G. *et al.* Spectrum of infection and risk factors for human Monkeypox, United States, 2003. *Emerg. Infect. Dis.* **13,** 1332–9 (2007).

429. Croft, D. R. *et al.* Occupational risks during a Monkeypox outbreak, Wisconsin, 2003. *Emerg. Infect. Dis.* **13,** 1150–7 (2007).

430. Reynolds, M. G. *et al.* Clinical manifestations of human Monkeypox influenced by route of infection. *J. Infect. Dis.* **194,** 773–80 (2006).

431. Huhn, G. D. *et al.* Clinical characteristics of human Monkeypox, and risk factors for severe disease. *Clin. Infect. Dis.* **41,** 1742–51 (2005).

432. Heymann, D. L., Szczeniowski, M. & Esteves, K. Re-emergence of Monkeypox in Africa: a review of the past six years. *Br. Med. Bull.* **54,** 693–702 (1998).

433. Gispen, R., Brand-Saathof, B. B. & Hekker, a C. Monkeypox-specific antibodies in human and simian sera from the Ivory Coast and Nigeria. *Bull. World Health Organ.* **53,** 355–60 (1976).

434. Foster, S. *et al.* Human Monkeypox. *Bull. Wildl. Heal. Org* 569–576 (1972).

435. Learned, L. A. *et al.* Extended interhuman transmission of Monkeypox in a hospital community in the Republic of the Congo, 2003. *Am. J. Trop. Med. Hyg.* **73,** 428–34 (2005).

436. Arita, I. & Henderson, D. A. Smallpox and Monkeypox in non-human primates. *Bull. World Health Organ.* **39,** 277–83 (1968).

437. Rhodes, L. V *et al.* Hantavirus pulmonary syndrome associated with Monongahela virus, Pennsylvania. *Emerg. Infect. Dis.* **6,** 616–21 (2000).

438. Kariwa, H., Yoshimatsu, K. & Arikawa, J. Hantavirus infection in East Asia. *Comp. Immunol. Microbiol. Infect. Dis.* **30,** 341–56 (2007).

439. Douglas, M. W. *et al.* Murray Valley encephalitis in an adult traveller complicated by long-term flaccid paralysis: case report and review of the literature. *Trans. R. Soc. Trop. Med. Hyg.* **101,** 284–8 (2007).

440. Wong, S. H., Smith, D. W., Fallon, M. J. & Kermode, a G. Murray Valley encephalitis mimicking Herpes simplex encephalitis. *J. Clin. Neurosci.* **12,** 822–4 (2005).

441. Broom, A. K., Lindsay, M. D. a, Wright, A. E., Smith, D. W. & Mackenzie, J. S. Epizootic activity of Murray Valley encephalitis and Kunjin viruses in an aboriginal community in the southeast Kimberley region of Western Australia: results of mosquito fauna and virus isolation studies. *Am. J. Trop. Med. Hyg.* **69,** 277–83 (2003).

442. Stich, A. *et al.* Clinical and laboratory findings on the first imported case of Murray Valley encephalitis in Europe. *Clin. Infect. Dis.* **37,** e19–21 (2003).

443. Gordon, A. N. *et al.* Confirmed case of encephalitis caused by Murray Valley encephalitis virus infection in a horse. *J. Vet. Diagn. Invest.* **24,** 431–6 (2012).

444. Field, H. E. Bats and emerging zoonoses: Henipaviruses and SARS. *Zoonoses Public Health* **56,** 278–84 (2009).

445. Halpin, K., Young, P. L., Field, H. E. & Mackenzie, J. S. Isolation of Hendra virus from pteropid bats: a natural reservoir of Hendra virus. *J. Gen. Virol.* **81,** 1927–32 (2000).

446. Chadha, M. S. *et al.* Nipah Virus-associated Encephalitis. *Emerg. Infect. Dis.* **12,** 235–240 (2006).

447. Sendow, I. *et al.* Screening for Nipah virus infection in West Kalimantan province, Indonesia. *Zoonoses Public Health* **57,** 499–503 (2010).

448. Breed, A. C. *et al.* Prevalence of Henipavirus and Rubulavirus antibodies in Pteropid bats, Papua New Guinea. *Emerg. Infect. Dis.* **16,** 1997–9 (2010).

449. Li, Y. Y. *et al.* Antibodies to Nipah or Nipah-like viruses in bats, China. *Emerg. Infect. Dis.* **14,** 1974–1976 (2008).

450. Drexler, J. F. *et al.* Henipavirus RNA in African bats. *PLoS One* **4,** e6367 (2009).

451. Shirai, J. *et al.* Nipah virus survey of flying foxes in Malaysia. *Japan Agric. Res. Q.* **41,** 69–78 (2007).

452. Lam, S.-K. Nipah virus--a potential agent of bioterrorism? *Antiviral Res.* **57,** 113–9 (2003).

453. Nahar, N., Sultana, R., Gurley, E. S., Hossain, M. J. & Luby, S. P. Date palm sap collection: exploring opportunities to prevent Nipah transmission. *Ecohealth* **7,** 196–203 (2010).

454. Luby, S. P. *et al.* Foodborne transmission of Nipah virus, Bangladesh. *Emerg. Infect. Dis.* **12,** 1888–94 (2006).

455. Chadha, M. S. *et al.* Nipah virus-associated encephalitis outbreak, Siliguri, India. *Emerg. Infect. Dis.* **12,** 235–40 (2006).

456. Chua, K. B. Nipah virus outbreak in Malaysia. *J. Clin. Virol.* **26,** 265–75 (2003).

457. Chan, K. P. *et al.* A survey of Nipah virus infection among various risk groups in Singapore. *Epidemiol. Infect.* **128,** 93–8 (2002).

458. Chua, K. B. *et al.* Isolation of Nipah virus from Malaysian Island flying-foxes. *Microbes Infect.* **4,** 145–51 (2002).

459. Ali, R. Nipah Virus Infection Among Military Personnel Involved in Pig Culling during an Outbreak of Encephalitis in Malaysia, 1998-1999. *Emerg. Infect. Dis.* **7,** 759–761 (2001).

460. Parashar, U. D. *et al.* Case-control study of risk factors for human infection with a new zoonotic paramyxovirus, Nipah virus, during a 1998-1999 outbreak of severe encephalitis in Malaysia. *J. Infect. Dis.* **181,** 1755–9 (2000).

461. Rahman, S. A. *et al.* Characterization of Nipah virus from naturally infected *Pteropus vampyrus* bats, Malaysia. *Emerg. Infect. Dis.* **16,** 1990–1993 (2010).

462. Chew, M. H. *et al.* Risk factors for Nipah virus infection among abattoir workers in Singapore. *J. Infect. Dis.* **181,** 1760–3 (2000).

463. Homaira, N. *et al.* Cluster of Nipah virus infection, Kushtia District, Bangladesh, 2007. *PLoS One* **5,** e13570 (2010).

464. Homaira, N. *et al.* Nipah virus outbreak with person-to-person transmission in a district of Bangladesh, 2007. *Epidemiol. Infect.* **138,** 1630–6 (2010).

465. Siva, S. R. *et al.* Ten year clinical and serological outcomes of Nipah virus infection. *Neurol. Asia* **14,** 53 – 58 (2009).

466. Luby, S. P. *et al.* Recurrent zoonotic transmission of Nipah virus into humans, Bangladesh, 2001-2007. *Emerg. Infect. Dis.* **15,** 1229–35 (2009).

467. Lo, M. K. & Rota, P. A. The emergence of Nipah virus, a highly pathogenic Paramyxovirus. *J. Clin. Virol.* **43,** 396–400 (2008).

468. Montgomery, J. M. *et al.* Risk factors for Nipah virus encephalitis in Bangladesh. *Emerg. Infect. Dis.* **14,** 1526–1532 (2008).

469. Lin, D. *et al.* Analysis of the complete genome of the tick-borne Flavivirus Omsk hemorrhagic fever virus. *Virology* **313,** 81–90 (2003).

470. Růžek, D., Yakimenko, V. V, Karan, L. S. & Tkachev, S. E. Omsk haemorrhagic fever. *Lancet* **376,** 2104–13 (2010).

471. Holbrook, M. R. *et al.* An animal model for the tickborne Flavivirus--Omsk hemorrhagic fever virus. *J. Infect. Dis.* **191,** 100–8 (2005).

472. Bessaud, M. *et al.* O’nyong-nyong Virus, Chad. *Emerg. Infect. Dis.* **12,** 1248–50 (2006).

473. Johnson, B. K. *et al.* Recovery of O’nyong-Nyong virus from *Anopheles funestus* in Western Kenya. *Trans. R. Soc. Trop. Med. Hyg.* **75,** 239–41 (1981).

474. Sanders, E. J. *et al.* O’nyong-nyong fever in south-central Uganda, 1996-1997: description of the epidemic and results of a household-based seroprevalence survey. *J. Infect. Dis.* **180,** 1436–43 (1999).

475. Kuniholm, M. H. *et al.* Seroprevalence and distribution of Flaviviridae, Togaviridae, and Bunyaviridae arboviral infections in rural Cameroonian adults. *Am. J. Trop. Med. Hyg.* **74,** 1078–83 (2006).

476. Ünal, G. & Gündefi, S. Human Orf : Echtyma Contagiosum Report of Five Cases. *TURKISH J. Med. Sci.* **32,** 173–175 (2002).

477. Inoshima, Y., Ito, M. & Ishiguro, N. Spatial and temporal genetic homogeneity of orf viruses infecting Japanese Serows *(Capricornis crispus)*. *J. Vet. Med. Sci.* **72,** 701–7 (2010).

478. Frandsen, J., Enslow, M. & Bowen, A. R. Orf parapoxvirus infection from a cat scratch. *Dermatol. Online J.* **17,** (2011).

479. Lojkic, I. *et al.* Phylogenetic analysis of Croatian Orf viruses isolated from sheep and goats. *Virol. J.* **7,** 314 (2010).

480. Klein, J. & Tryland, M. Characterisation of parapoxviruses isolated from Norwegian semi-domesticated reindeer *(Rangifer tarandus tarandus)*. *Virol. J.* **2,** 79 (2005).

481. Mazur, C., Ferreira, I. I., Rangel Filho, F. B. & Galler, R. Molecular characterization of Brazilian isolates of orf virus. *Vet. Microbiol.* **73,** 253–9 (2000).

482. Mondal, B., Bera, A. K., Hosamani, M., Tembhurne, P. A. & Bandyopadhyay, S. K. Detection of Orf virus from an outbreak in goats and its genetic relation with other parapoxviruses. *Vet. Res. Commun.* **30,** 531–9 (2006).

483. Vikøren, T. *et al.* A severe outbreak of contagious ecthyma (Orf) in a free-ranging musk ox *(Ovibos moschatus)* population in Norway. *Vet. Microbiol.* **127,** 10–20 (2008).

484. Tryland, M., Josefsen, T. D., Oksanen, A. & Aschfalk, A. Parapoxvirus infection in *(Norwegian tarandus tarandus )* Factors affecting the outcome in dogs with multicentric. *Vet. Rec.* 394–396 (2001).

485. Scagliarini, a *et al.* Characterisation of immunodominant protein encoded by the F1L gene of orf virus strains isolated in Italy. *Arch. Virol.* **147,** 1989–95 (2002).

486. Oem, J.-K. *et al.* Phylogenetic analysis and characterization of Korean orf virus from dairy goats: case report. *Virol. J.* **6,** 167 (2009).

487. Obi, T. U. & Gibbs, E. P. J. Orf in sheep and goats in Nigeria. *Trop. Anim. Health Prod.* **10,** 233–235 (1978).

488. Azwai, S. M., Carter, S. D. & Woldehiwet, Z. Immune responses of the camel (Camelus dromedarius) to contagious ecthyma (Orf) virus infection. *Vet. Microbiol.* **47,** 119–31 (1995).

489. Chan, K.-W. *et al.* Differential diagnosis of orf viruses by a single-step PCR. *J. Virol. Methods* **160,** 85–9 (2009).

490. Chan, K.-W. *et al.* Identification and phylogenetic analysis of Orf virus from goats in Taiwan. *Virus Genes* **35,** 705–12 (2007).

491. De la Concha-Bermejillo, A., Guo, J., Zhang, Z. & Waldron, D. Severe persistent Orf in young goats. *J. Vet. Diagnostic Investig.* **15,** 423–431 (2003).

492. Hosamani, M. *et al.* Isolation and characterization of an Indian Orf virus from goats. *Zoonoses Public Health* **54,** 204–8 (2007).

493. Terzian, A. C. B. *et al.* Sporadic Oropouche virus infection, Acre, Brazil. *Emerg. Infect. Dis.* **15,** 348–350 (2009).

494. Nunes, M. R. T. *et al.* Oropouche virus isolation, southeast Brazil. *Emerg. Infect. Dis.* **11,** 1610–3 (2005).

495. Mercer, D. R. & Castillo-pizango, M. J. Changes in relative species compositions of biting midges (Diptera: Ceratopogonidae) and an outbreak of Oropouche virus in Iquitos, Peru. *Entomol. Soc. Am.* **42,** 554–558 (2005).

496. Ebel, G. D. Update on Powassan virus: emergence of a North American tick-borne Flavivirus. *Annu. Rev. Entomol.* **55,** 95–110 (2010).

497. Leonova, G. N. *et al.* Characterization of Powassan viruses from Far Eastern Russia. *Arch. Virol.* **154,** 811–20 (2009).

498. Brackney, D. E., Nofchissey, R. A., Fitzpatrick, K. A., Brown, I. K. & Ebel, G. D. Short Report: Stable prevalence of Powassan virus in *Ixodes scapularis* in a Northern Wisconsin focus. *Am. J. Trop. Med. Hyg.* **79,** 971–973 (2008).

499. Zarnke, L. & Yuill, M. Powassan virus infection in snowshoe hares *(Lepus americanus)*. *J. Wildl. Dis.* **17,** (1981).

500. Johnson, H. N. Isolation of Powassan virus from a spotted skunk in California. *J. Wildl. Dis.* **23,** (1987).

501. Abubakr, M. I. *et al.* Pseudocowpox virus: the etiological agent of contagious ecthyma (Auzdyk) in camels *(Camelus dromedarius)* in the Arabian peninsula. *Vector borne zoonotic Dis.* **7,** 257–60 (2007).

502. Tikkanen, M. K. *et al.* Recent isolates of Parapoxvirus of Finnish reindeer *(Rangifer tarandus tarandus)* are closely related to bovine pseudocowpox virus. *J. Gen. Virol.* **85,** 1413–1418 (2004).

503. Asagba, M. O. Recognition of pseudocowpox in Nigeria. *Trop. Anim. Health Prod.* **14,** 2000 (1982).

504. MacNeil, a *et al.* Diagnosis of bovine-associated parapoxvirus infections in humans: molecular and epidemiological evidence. *Zoonoses Public Health* **57,** e161–4 (2010).

505. Hofmann, J. *et al.* Hantavirus outbreak, Germany, 2007. *Emerg. Infect. Dis.* **14,** 381–90 (2008).

506. Schilling, S. *et al.* Hantavirus disease outbreak in Germany: limitations of routine serological diagnostics and clustering of virus sequences of human and rodent origin. *J. Clin. Microbiol.* **45,** 3008–14 (2007).

507. Abu Sin, M. *et al.* Risk factors for Hantavirus infection in Germany, 2005. *Emerg. Infect. Dis.* **13,** 1364–6 (2007).

508. Essbauer, S. *et al.* A new Puumala hantavirus subtype in rodents associated with an outbreak of Nephropathia epidemica in South-East Germany in 2004. *Epidemiol. Infect.* **134,** 1333–44 (2006).

509. Mailles, A. *et al.* Increase of Hantavirus infections in France, 2003. *Médecine Mal. Infect.* **35,** 68–72 (2005).

510. Hukic, M. *et al.* A serosurvey reveals Bosnia and Herzegovina as a Europe’s hotspot in Hantavirus seroprevalence. *Epidemiol. Infect.* **138,** 1185–93 (2010).

511. Winter, C. H. *et al.* Survey and case-control study during epidemics of Puumala virus infection. *Epidemiol. Infect.* **137,** 1479–85 (2009).

512. Schwarz, A. C., Ranft, U., Piechotowski, I., Childs, J. E. & Brockmann, S. O. Risk factors for human infection with Puumala virus, southwestern Germany. *Emerg. Infect. Dis.* **15,** 1032–9 (2009).

513. Mertens, M. *et al.* Seroepidemiological study in a Puumala virus outbreak area in South-East Germany. *Med. Microbiol. Immunol.* **198,** 83–91 (2009).

514. Pettersson, L., Boman, J., Juto, P., Evander, M. & Ahlm, C. Outbreak of Puumala virus infection, Sweden. *Emerg. Infect. Dis.* **14,** 808–10 (2008).

515. Cvetko, L. *et al.* Puumala virus in Croatia in the 2002 HFRS outbreak. *J. Med. Virol.* **77,** 290–4 (2005).

516. Escutenaire, S. *et al.* Evidence of Puumala Hantavirus infection in red foxes *(Vulpes vulpes)* in Belgium. *Vet. Rec.* **147,** 365–6 (2000).

517. Uyar, Y., Caglayik, D. Y., Korukluo, G., A., C. & Ertek, M. Hantavirus cases among CCHFv-PCR and ELISA negative patients in Hantavirus outbreak area, in Turkey. *J. Clin. Virol.* **46,** s15–s61 (2009).

518. Braun, N. *et al.* Characterization and outcome following Puumala virus infection: a retrospective analysis of 75 cases. *Nephrol. Dial. Transplant.* **25,** 2997–3003 (2010).

519. Hjertqvist, M., Klein, S. L., Ahlm, C. & Klingstrom, J. Mortality Rate Patterns for Hemorrhagic Fever with Renal Syndrome Caused by Puumala Virus. *Emerg. Infect. Dis.* **16,** 1584–1586 (2010).

520. Makary, P. *et al.* Disease burden of Puumala virus infections, 1995-2008. *Epidemiol. Infect.* **138,** 1484–92 (2010).

521. Linard, C., Tersago, K., Leirs, H. & Lambin, E. F. Environmental conditions and Puumala virus transmission in Belgium. *Int. J. Health Geogr.* **6,** 55 (2007).

522. Warrell, M. & Warrell, D. Rabies and other Lyssavirus diseases. *Lancet* **363,** 959–69 (2004).

523. Favi, M. *et al.* First case of human rabies in Chile caused by an insectivorous bat virus variant. *Emerg. Infect. Dis.* **8,** 79–81 (2002).

524. Blanton, J. D., Palmer, D. & Rupprecht, C. E. Rabies surveillance in the United States during 2009. *J. Am. Med. Assoc.* **237,** 646–657 (2010).

525. Blanton, J. D., Krebs, J. W., Hanlon, C. A. & Rupprecht, C. E. Rabies surveillance in the United States during 2005. *J. Am. Med. Assoc.* **229,** 1897–1911 (2006).

526. Krebs, J. W., Wheeling, J. T. & Childs, J. E. Rabies surveillance in the United States during 2002. *J. Am. Vet. Med. Assoc.* **223,** 1736–1748 (2003).

527. Badillo, R., Mantilla, J. C. & Pradilla, G. Encefalitis rábica humana por mordedura de murciélago en un área urbana de Colombia. *Biomedica* **29,** 191–203 (2009).

528. Blanton, J. D., Robertson, K., Palmer, D. & Rupprecht, C. E. Rabies surveillance in the United States during 2008. *J. Am. Med. Assoc.* **235,** 676–689 (2009).

529. Blanton, J. D., Palmer, D., Christian, K. A., Rupprecht, C. E. & D, P. Rabies surveillance in the United States during 2007. *J. Am. Med. Assoc.* **233,** 884–897 (2008).

530. Blanton, J. D., Hanlon, C. A. & Rupprecht, C. E. Rabies surveillance in the United States during 2006. *J. Am. Med. Assoc.* **231,** 540–556 (2007).

531. Krebs, J. W., Noll, H. R., Rupprecht, C. E. & Childs, J. E. Rabies surveillance in the United States during 2001. *J. Am. Vet. Med. Assoc.* **221,** (2002).

532. Kuzmin, I. *et al.* Bat lyssaviruses (Aravan and Khujand) from Central Asia: phylogenetic relationships according to N, P and G gene sequences. *Virus Res.* **97,** 65–79 (2003).

533. Morikawa, S., Saijo, M. & Kurane, I. Current knowledge on lower virulence of Reston Ebola virus (in French: Connaissances actuelles sur la moindre virulence du virus Ebola Reston). *Comp. Immunol. Microbiol. Infect. Dis.* **30,** 391–8 (2007).

534. Barrette, R. W. *et al.* Discovery of swine as a host for the Reston ebolavirus. *Science* **325,** 204–6 (2009).

535. Morris, K. First pig-to-human transmission of Ebola-Reston virus. *Lancet Infect. Dis.* **9,** 148–148 (2009).

536. Report, W. Ebola haemorrhagic fever in Sudan, 1976. *Bull. World Health Organ.* **56,** (1978).

537. Xu, F., Chen, H., Travassos da Rosa, A. P. a, Tesh, R. B. & Xiao, S.-Y. Phylogenetic relationships among Sandfly fever group viruses (Phlebovirus: Bunyaviridae) based on the small genome segment. *J. Gen. Virol.* **88,** 2312–9 (2007).

538. Pourrut, X. *et al.* Rift Valley fever virus seroprevalence in human rural populations of Gabon. *PLoS Negl. Trop. Dis.* **4,** e763 (2010).

539. Sissoko, D. *et al.* Rift Valley Fever, Mayotte, 2007–2008. *Emerg. Infect. Dis.* **15,** 568–570 (2009).

540. Faye, O. *et al.* Rift Valley fever outbreak with East-Central African virus lineage in Mauritania, 2003. *Emerg. Infect. Dis.* **13,** 1016–23 (2007).

541. LaBeaud, A. D., Ochiai, Y., Peters, C. J., Muchiri, E. M. & King, C. H. Spectrum of Rift Valley fever virus transmission in Kenya: insights from three distinct regions. *Am. J. Trop. Med. Hyg.* **76,** 795–800 (2007).

542. Abdo-Salem, S. *et al.* Descriptive and spatial epidemiology of Rift valley fever outbreak in Yemen 2000-2001. *Ann. N. Y. Acad. Sci.* **1081,** 240–242 (2006).

543. Balkhy, H. H. & Memish, Z. a. Rift Valley fever: an uninvited zoonosis in the Arabian peninsula. *Int. J. Antimicrob. Agents* **21,** 153–7 (2003).

544. Evans, A. *et al.* Prevalence of antibodies against Rift Valley fever virus in Kenyan wildlife. *Epidemiol. Infect.* **136,** 1261–9 (2008).

545. Andriamandimby, S. F. *et al.* Rift Valley Fever during rainy seasons, Madagascar, 2008 and 2009. *Emerg. Infect. Dis.* **16,** 963–970 (2010).

546. Bird, B. H. & Ksiazek, T. G. Zoonosis Update Rift Valley fever virus. *J. Am. Med. Assoc.* **234,** 883–893 (2009).

547. Tong, S. Ross River virus disease in Australia: epidemiology, socioecology and public health response. *Intern. Med. J.* **34,** 58–60 (2004).

548. Kelly-hope, A. L. A., Purdie, D. M., Kay, B. H. & Kelly-hope, L. A. Ross River virus disease in Australia, 1886 – 1998, with analysis of risk factors associated with outbreaks. *Entomol. Soc. Am.* **41,** 133–150 (2004).

549. Klapsing, P. *et al.* Ross River virus disease reemergence, Fiji. *Emerg. Infect. Dis.* **11,** 2003–2004 (2005).

550. Tappe, D. *et al.* Ross River virus infection in a traveller returning from northern Australia. *Med. Microbiol. Immunol.* **198,** 271–3 (2009).

551. Hossain, I., Tambyah, P. A. & Wilder-Smith, A. Ross River virus disease in a traveler to Australia. *J. Travel Med.* **16,** 420–3 (2009).

552. Harley, D., Sleigh, A. & Ritchie, S. Ross River virus transmission, infection, and disease: a cross-disciplinary review. *Clin. Microbiol. Rev.* **14,** 909–32, table of contents (2001).

553. Bányai, K. *et al.* Whole genome sequencing and phylogenetic analysis of a zoonotic human G8P[14] Rotavirus strain. *Infect. Genet. Evol.* **10,** 1140–4 (2010).

554. Nokes, D. J. *et al.* Incidence and clinical characteristics of group A Rotavirus infections among children admitted to hospital in Kilifi, Kenya. *PLoS Med.* **5,** (2008).

555. Rodríguez-Limas, W. a, Flores-Samaniego, B., de la Mora, G., Ramírez, O. T. & Palomares, L. a. Genotypification of bovine group A Rotavirus in México. *Vaccine* **27,** 6411–4 (2009).

556. Alfieri, A. A., Parazzi, M. E., Takiuchi, E., Médici, K. C. & Alfieri, A. F. Frequency of group A rotavirus in diarrhoeic calves in Brazilian cattle herds, 1998–2002. *Trop. Anim. Health Prod.* **38,** 521–526 (2006).

557. Jain, V. *et al.* Great diversity of group A Rotavirus strains and high prevalence of mixed Rotavirus infections in India. *J. Clin. Microbiol.* **39,** (2001).

558. Mas Marques, A., Diedrich, S., Huth, C. & Schreier, E. Group A Rotavirus genotypes in Germany during 2005/2006. *Arch. Virol.* **152,** 1743–9 (2007).

559. Collins, P. J., Martella, V., Sleator, R. D., Fanning, S. & O’Shea, H. Detection and characterisation of group A Rotavirus in asymptomatic piglets in southern Ireland. *Arch. Virol.* **155,** 1247–59 (2010).

560. Leung, A. K. C., Kellner, J. D. & Davies, H. D. Rotavirus gastroenteritis. *Adv. Ther.* **22,** 476–87 (2005).

561. Steyer, A. *et al.* Molecular analysis of human group A Rotavirus G10P[14] genotype in Slovenia. *J. Clin. Virol.* **49,** 121–5 (2010).

562. Fukai, K. *et al.* Molecular characterization of a novel bovine group A Rotavirus. *Vet. Microbiol.* **123,** 217–24 (2007).

563. Ghosh, S. *et al.* Molecular characterization of bovine group A Rotavirus G3P[3] strains. *Arch. Virol.* **152,** 1935–40 (2007).

564. Iturriza-gómara, M. *et al.* Molecular epidemiology of human group A Rotavirus infections in the United Kingdom between 1995 and 1998. *J. Clin. Microbiol.* **38,** (2000).

565. Page, N. *et al.* Emergence and characterization of serotype G9 rotavirus strains from Africa. *J. Infect. Dis.* **202 Suppl,** S55–63 (2010).

566. Unicomb, L. E. *et al.* Evidence of high-frequency genomic reassortment of group A Rotavirus strains in Bangladesh: emergence of type G9 in 1995. *J. Clin. Microbiol.* **37,** (1999).

567. Dionisio, D. *et al.* Encephalitits without meningitis due to Sandfly fever virus serotype Toscana. *Clin. Infect. Dis.* **32,** 1241–1243 (2001).

568. Carhan, A. *et al.* Characterization of a Sandfly fever Sicilian virus isolated during a Sandfly fever epidemic in Turkey. *J. Clin. Virol.* **48,** 264–9 (2010).

569. Çarhan, A. *et al.* Characterization of a new Sandfly fever virus isolated during the 2008 Sandfly fever epidemic in Turkey. *J. Clin. Virol.* **46,** S1 – S14 (2009).

570. Ergünay, K., Saygan, M. B., Aydoğan, S., Pınar, A. & Us, D. Detection of Sandfly fever virus (SFV) igg antibodies in blood donors from Ankara, Turkey. *J. Clin. Virol.* **46,** S15 (2009).

571. Hukić, M. & Salimović-Besić, I. Sandfly - Pappataci fever in Bosnia and Herzegovina: the new-old disease. *Bosn. J. basic Med. Sci.* **9,** 39–43 (2009).

572. Ellis, S. B. *et al.* Outbreak of Sandfly fever in central Iraq, September 2007. *Mil. Med.* **173,** 949–53 (2008).

573. Izri, A. *et al.* Sandfly fever Sicilian virus, Algeria. *Emerg. Infect. Dis.* **14,** 795–798 (2008).

574. Papa, A., Konstantinou, G., Pavlidou, V. & Antoniadis, A. Sandfly fever virus outbreak in Cyprus. *Clin. Microbiol. Infect.* **12,** 192–4 (2006).

575. Dionisio, D., Esperti, F., Vivarelli, A. & Valassina, M. Epidemiological, clinical and laboratory aspects of Sandfly fever. *Curr. Opin. Infect. Dis.* **16,** 383–8 (2003).

576. Brett-Major, D. M. & Claborn, D. M. Sandfly fever: what have we learned in one hundred years? *Mil. Med.* **174,** 426–31 (2009).

577. Rihtaric, D., Hostnik, P., Steyer, A., Grom, J. & Toplak, I. Identification of SARS-like Coronaviruses in horseshoe bats *(Rhinolophus hipposideros)* in Slovenia. *Arch. Virol.* **155,** 507–14 (2010).

578. Tong, S. *et al.* Detection of novel SARS-like and other Coronaviruses in bats from Kenya. *Emerg. Infect. Dis.* **15,** 482–485 (2009).

579. Shi, Z. & Hu, Z. A review of studies on animal reservoirs of the SARS coronavirus. *Virus Res.* **133,** 74–87 (2008).

580. Wang, L. F. & Eaton, B. T. Bats, civets and the emergence of SARS. *Curr. Top. Microbiol. Immunol.* **315,** 325–44 (2007).

581. Wang, L.-F. *et al.* Review of bats and SARS. *Emerg. Infect. Dis.* **12,** 1834–40 (2006).

582. Wang, M. *et al.* SARS-CoV infection in a restaurant from palm civet. *Emerg. Infect. Dis.* **11,** 1860–5 (2005).

583. Clark, C., McIntyre, P. G., Evans, A., McInnes, C. J. & Lewis-Jones, S. Human Sealpox resulting from a seal bite: confirmation that Sealpox virus is zoonotic. *Br. J. Dermatol.* **152,** 791–3 (2005).

584. Becher, P., König, M., Müller, G., Siebert, U. & Thiel, H.-J. Characterization of Sealpox virus, a separate member of the Parapoxviruses. *Arch. Virol.* **147,** 1133–40 (2002).

585. Tryland, M., Klein, J., Nordøy, E. S. & Blix, a S. Isolation and partial characterization of a Parapoxvirus isolated from a skin lesion of a Weddell seal. *Virus Res.* **108,** 83–7 (2005).

586. Hicks, B. D. & Worthy, G. A. J. Sealpox in captive grey seals *(Halichoerus grypus)* and their handlers. *J. Wildl. Dis.* **23,** (1987).

587. Wilson, T. M., Booth, A. D. & Chevillej, N. F. Sealpoix field survey. *J. Wildl. Dis.* **8,** 158–160 (1972).

588. Plyusnina, A. *et al.* Identification of Seoul Hantavirus in *Rattus norvegicus* in Indonesia. *Scand. J. Infect. Dis.* **36,** 356–359 (2004).

589. Johansson, P. *et al.* Molecular characterization of two Hantavirus strains from different *Rattus* species in Singapore. *Virol. J.* **7,** 1–9 (2010).

590. Nielsen, C. F. *et al.* Seoul virus infection in a Wisconsin patient with recent travel to China, March 2009: first documented case in the Midwestern United States. *Am. J. Trop. Med. Hyg.* **83,** 1266–8 (2010).

591. Zhang, Y.-Z. *et al.* Seoul Virus and Hantavirus Disease, Shenyang, People’s Republic of China. *Emerg. Infect. Dis.* **15,** 200–206 (2009).

592. Zuo, S. *et al.* Seoul virus in patients and rodents from Beijing, China. *Am. J. Trop. Med. Hyg.* **78,** 833–7 (2008).

593. Heyman, P. *et al.* Seoul Hantavirus in Europe: first demonstration of the virus genome in wild *Rattus norvegicus* captured in France. *Eur. J. Clin. Microbiol. Infect. Dis.* **23,** 711–7 (2004).

594. Reynes, J.-M. *et al.* Evidence of the presence of Seoul virus in Cambodia. *Microbes Infect.* **5,** 769–773 (2003).

595. Lokugamage, K. *et al.* Genetic and antigenic characterization of the Amur virus associated with hemorrhagic fever with renal syndrome. *Virus Res.* **101,** 127–34 (2004).

596. Jiang, J., Zhang, W., Wu, X., Zhang, P. & Cao, W. Soochong virus and Amur virus might be the same entities of Hantavirus. *J. Med. Virol.* **79,** 1792–1795 (2007).

597. Liu, W., Worobey, M., Li, Y. & Keele, B. Molecular ecology and natural history of Simian foamy virus infection in wild-living chimpanzees. *PLoS Pathog.* **4,** e1000097 (2008).

598. Mouinga-Ondémé, A. *et al.* Two distinct variants of Simian foamy virus in naturally infected mandrills *(Mandrillus sphinx)* and cross-species transmission to humans. *Retrovirology* **7,** 105 (2010).

599. Sandstrom, P. A. *et al.* Simian foamy virus infection among zoo keepers. *Lancet* **355,** 551–2 (2000).

600. Jones-Engel, L. *et al.* Diverse contexts of zoonotic transmission of Simian foamy viruses in Asia. *Emerg. Infect. Dis.* **14,** 1200–1208 (2008).

601. Switzer, W. M. *et al.* Coinfection with HIV-1 and Simian foamy virus in west central Africans. *J. Infect. Dis.* **197,** 1389–93 (2008).

602. Boneva, R. S. *et al.* Clinical and virological characterization of persistent human infection with Simian foamy viruses. *AIDS Res. Hum. Retroviruses* **23,** 1330–7 (2007).

603. Calattini, S. *et al.* Simian foamy virus transmission from apes to humans, rural Cameroon. *Emerg. Infect. Dis.* **13,** 1314–1320 (2007).

604. Cooke, F. J. & Shapiro, D. S. First report of Simian foamy virus in Asia. *Int. J. Infect. Dis.* **9,** 237–238 (2005).

605. Switzer, W. M. *et al.* Frequent Simian foamy virus infection in persons occupationally exposed to nonhuman primates. *J. Virol.* **78,** 2780–2789 (2004).

606. VandeWoude, S. & Apetrei, C. Going wild: lessons from naturally occurring T-lymphotropic lentiviruses. *Clin. Microbiol. Rev.* **19,** 728–762 (2006).

607. Switzer, W. M. *et al.* The epidemiology of simian immunodeficiency virus infection in a large number of wild- and captive-born chimpanzees: evidence for a recent introduction following chimpanzee divergence. *AIDS Res. Hum. Retroviruses* **21,** 335–42 (2005).

608. Rudicell, R. S. *et al.* Impact of simian immunodeficiency virus infection on chimpanzee population dynamics. *PLoS Pathog.* **6,** (2010).

609. Neel, C. *et al.* Molecular epidemiology of simian immunodeficiency virus infection in wild-living gorillas. *J. Virol.* **84,** 1464–76 (2010).

610. Santiago, M. L. *et al.* Simian Immunodeficiency virus infection in free-ranging Sooty Mangabeys *(Cercocebus atys atys)* from the Taı Forest, Cote d’Ivoire: implications for the origin of epidemic Human Immunodeficiency virus Type 2. *Society* **79,** 12515–12527 (2005).

611. Takemura, T. *et al.* A novel simian immunodeficiency virus from black mangabey *(Lophocebus aterrimus)* in the Democratic Republic of Congo. *J. Gen. Virol.* **86,** 1967–71 (2005).

612. Nerrienet, E. *et al.* Simian Immunodeficiency virus infection in wild-caught chimpanzees from Cameroon. *J. Virol.* **79,** 1312–1319 (2005).

613. M, P. *et al.* Risk to human health from a plethora of Simian Immunodeficiency viruses in primate bushmeat. *Emerg. Infect. Dis.* **8,** 451–457 (2002).

614. Hara, M. *et al.* Isolation and characterization of a new simian retrovirus type D subtype from monkeys at the Tsukuba Primate Center, Japan. *Microbes Infect.* **7,** 126–31 (2005).

615. Meurphy, H. W. *et al.* Implications of Simian Retroviruses for captive primate population management and the occupational saftey of primate handlers. *J. Zoo Wildl. Med.* **37,** 219–233 (2006).

616. Lerche, N. W. *et al.* Evidence of infection with Simian Type D Retrovirus in persons occupationally exposed to nonhuman primates. *J. Virol.* **75,** 1783–1789 (2001).

617. Nandi, J. S., Bhavalkar-Potdar, V., Tikute, S. & Raut, C. G. A novel type D Simian Retrovirus naturally infecting the Indian Hanuman langur *(Semnopithecus entellus)*. *Virology* **277,** 6–13 (2000).

618. Imamova, L. R., Kzhyshkowska, Y. G., Ostashkin, a. S., Itkes, a. V. & Il’in, K. V. Human spontaneous laryngeal carcinoma HEp-2 cells are chronically infected with SRV-1 virus, a variant of simian type D retrovirus. *Bull. Exp. Biol. Med.* **129,** 584–586 (2000).

619. Rivers, M. N., Alexander, J. L., Rohde, R. E. & Pierce, J. R. Hantavirus pulmonary syndrome in Texas: 1993-2006. *South. Med. Assoc.* **102,** 36–41 (2009).

620. Webster, D. *et al.* Cluster of cases of Hantavirus pulmonary syndrome in Alberta, Canada. *Am. J. Trop. Med. Hyg.* **77,** 914–8 (2007).

621. Sinclair, J. R. *et al.* Two cases of Hantavirus pulmonary syndrome in Randolph County, West Virginia: a coincidence of time and place? *Am. J. Trop. Med. Hyg.* **76,** 438–42 (2007).

622. Cline, B. J., Carver, S. & Douglass, R. J. Relationship of human behavior within outbuildings to potential exposure to Sin Nombre virus in Western Montana. *Ecohealth* **7,** 389–393 (2010).

623. Gonzalez, L. M. *et al.* Prevalence of antibodies to Sin Nombre virus in humans living in rural areas of southern New Mexico and western Texas. *Virus Res.* **74,** 177–9 (2001).

624. Torres-Perez, F. *et al.* Sin Nombre virus infection in field workers, Colorado, USA. *Emerg. Infect. Dis.* **16,** 308–311 (2010).

625. Mattar, S. & Parra, M. Serologic evidence of Hantavirus infection in humans, Colombia. *Emerg. Infect. Dis.* **10,** 2263–2265 (2004).

626. Bayard, V. *et al.* Outbreak of hantavirus pulmonary syndrome, Los Santos, Panama, 1999-2000. *Emerg. Infect. Dis.* **10,** 1635–42 (2004).

627. Mendes, W. S. *et al.* Hantavirus Infection in Anajatuba Maranhao, Brazil. *Emerg. Infect. Dis.* **10,** 1496–1498 (2004).

628. Overturf, G. D. Clinical Sin Nombre Hantaviral Infections in Children. *Pediatr. Infect. Dis. J.* **24,** 373–374 (2005).

629. Calisher, C. H. *et al.* Epizootiology of Sin Nombre and El Moro Canyon hantaviruses, southeastern Colorado, 1995-2000. *J. Wildl. Dis.* **41,** 1–11 (2005).

630. Douglass, R. J., Kuenzi, A. J., Williams, C. Y., Douglass, S. J. & Mills, J. N. Removing deer mice from buildings and the risk for human exposure to Sin Nombre virus. *Emerg. Infect. Dis.* **9,** 390–392 (2003).

631. Ramos, M. M., Overturf, G. D., Crowley, M. R., Rosenberg, R. B. & Hjelle, B. Infection with Sin Nombre Hantavirus: clinical presentation and outcome in children and adolescents. *Pediatrics* **108,** e27–e27 (2001).

632. Passaro, D. J. *et al.* Preominant kidney involvemnet in a fatal case of Hantavirus pulmonary syndrom casued by Sin Nombre virus. *Clin. Infect. Dis.* **33,** 263–4 (2001).

633. Holsomback, T. S. *et al.* Bayou virus detected in non-Oryzomyine rodent hosts: an assessment of habitat composition, reservoir community structure, and marsh rice rat social dynamics. *J. Vector Ecol.* **34,** 9–21 (2009).

634. Pini, N. *et al.* Hantavirus Infection in Humans and Rodents, Northwestern Argentina. *Emerg. Infect. Dis.* **9,** 1070–1076 (2003).

635. Buckley, A. *et al.* Serological evidence of West Nile virus, Usutu virus and Sindbis virus infection of birds in the UK. *J. Gen. Virol.* **84,** 2807–2817 (2003).

636. Kurkela, S. *et al.* Sindbis virus infection in resident birds, migratory birds, and humans, Finland. *Emerg. Infect. Dis.* **14,** 41–7 (2008).

637. Luukkainen, R., Laine, M. & Nirhamo, J. Chronic arthritis after Sindbis-related (Pogosta) virus infection. *Scand. J. Rheumatol.* **29,** 399–400 (2000).

638. Brummer-Korvenkontio, M. *et al.* Epidemiology of Sindbis virus infections in Finland 1981-96: possible factors explaining a peculiar disease pattern. *Epidemiol. Infect.* **129,** 335–45 (2002).

639. Juricova, Z., Hubalek, Z., Halouzka, J. & Sikutova, S. Serological examination of songbirds (Passeriformes) for mosquito-borne viruses Sindbis, Tahyna, and Batai in a South Moravian wetland (Czech Republic). *Vector-borne zoonotic Dis.* **9,** 295–299 (2009).

640. Lundstrom, J. O. & Pfeffer, M. Phylogeographic structure and evolutionary history of Sindbis virus.pdf. *Vector-borne zoonotic Dis.* **10,** 889–907 (2010).

641. Pierro, D. J., Powers, E. L. & Olson, K. E. Genetic determinants of Sindbis virus mosquito infection are associated with a highly conserved Alphavirus and Flavivirus envelope sequence. *J. Virol.* **82,** 2966–74 (2008).

642. Sammels, L. M., Lindsay, M. D., Poidinger, M., Coelen, R. J. & Mackenzie, J. S. Geographic distribution and evolution of Sindbis virus in Australia. *J. Gen. Virol.* **80 ( Pt 3),** 739–48 (1999).

643. Medlock, J. M., Snow, K. R. & Leach, S. Possible ecology and epidemiology of medically important mosquito-borne arboviruses in Great Britain. *Epidemiol. Infect.* **135,** 466–82 (2007).

644. Medeiros, D. B. A., Nunes, M. R. T., Vasconcelos, P. F. C., Chang, G.-J. J. & Kuno, G. Complete genome characterization of Rocio virus (Flavivirus: Flaviviridae), a Brazilian Flavivirus isolated from a fatal case of encephalitis during an epidemic in Sao Paulo state. *J. Gen. Virol.* **88,** 2237–46 (2007).

645. Day, J. F. & Shaman, J. Severe winter freezes enhance St. Louis encephalitis virus amplification and epidemic transmission in Peninsular Florida. *J. Med. Entomol.* **46,** 1498–1506 (2009).

646. Spinsanti, L. I. *et al.* Human outbreak of St. Louis encephalitis detected in Argentina, 2005. *J. Clin. Virol.* **42,** 27–33 (2008).

647. Wootton, S. H., Kaplan, S. L., Perrotta, D. M., Martin, D. A. & Campbell, G. L. St. Louis encephalitis in early infancy. *Pediatr. Infect. Dis. J.* **23,** 951–954 (2004).

648. Shaman, J., Day, J. F. & Stieglitz, M. The spatial-temporal distribution of drought, wetting, and human cases of St. Louis encephalitis in southcentral Florida. *Am. J. Trop. Med. Hyg.* **71,** 251–61 (2004).

649. Spinsanti, L. *et al.* St. Louis encephalitis in Argentina: The first case reported in the last seventeen years. *Emerg. Infect. Dis.* **9,** 271–273 (2003).

650. Wasay, M. *et al.* St Louis encephalitis. *Magn. Reson. Imaging* **57,** 114–118 (2000).

651. Rodrigues, S. G. *et al.* Molecular epidemiology of Saint Louis encephalitis virus in the Brazilian Amazon: genetic divergence and dispersal. *J. Gen. Virol.* **91,** 2420–7 (2010).

652. Gruwell, J. a *et al.* Role of peridomestic birds in the transmission of St. Louis encephalitis virus in southern California. *J. Wildl. Dis.* **36,** 13–34 (2000).

653. Hukkanen, R. R. *et al.* West Nile and St. Louis encephalitis virus antibody seroconversion, prevalence, and persistence in naturally infected pig-tailed macaques *(Macaca nemestrina)*. *Clin. Vaccine Immunol.* **13,** 711–4 (2006).

654. Trevejo, R. T. Acute encephalitis hospitalizations, California, 1990-1999: unrecognized Arboviral Encephalitis? *Emerg. Infect. Dis.* **10,** 1442–1449 (2004).

655. Reisen, W. K., Chiles, R. E., Martinez, V. M., Fang, Y. & Green, E. N. Encephalitis virus persistence in California birds: Experimental infections in mourning doves *(Zenaidura macroura)*. *J. Med. Entomol.* **41,** 462–466 (2004).

656. Lamunu, M. *et al.* Containing a haemorrhagic fever epidemic: the Ebola experience in Uganda (October 2000–January 2001). *Int. J. Infect. Dis.* **8,** 27–37 (2004).

657. Pourrut, X. *et al.* The natural history of Ebola virus in Africa. *Microbes Infect.* **7,** 1005–14 (2005).

658. Okware, S. I. *et al.* An outbreak of Ebola in Uganda. *Trop. Med. Int. Health* **7,** 1068–75 (2002).

659. Leroy, E. M. *et al.* A serological survey of Ebola virus infection in central African nonhuman primates. *J. Infect. Dis.* **190,** 1895–9 (2004).

660. Groseth, A., Feldmann, H. & Strong, J. E. The ecology of Ebola virus. *Trends Microbiol.* **15,** 408–16 (2007).

661. Leroy, E. M. *et al.* Human Ebola outbreak resulting from direct exposure to fruit bats in Luebo, Democratic Republic of Congo, 2007. *Vector Borne Zoonotic Dis.* **9,** 723–8 (2009).

662. Lu, Z. *et al.* Tahyna virus and human infection, China. *Emerg. Infect. Dis.* **15,** 306–309 (2009).

663. Gould, E. A., Higgs, S., Buckley, A. & Gritsun, T. S. Potential arbovirus emergence and implications for the United Kingdom. *Emerg. Infect. Dis.* **12,** 549–55 (2006).

664. Kilian, P., Růzek, D., Danielová, V., Hypsa, V. & Grubhoffer, L. Nucleotide variability of Tahyna virus (Bunyaviridae, Orthobunyavirus) small (S) and medium (M) genomic segments in field strains differing in biological properties. *Virus Res.* **149,** 119–23 (2010).

665. Butenko, A. M., Vladimirtseva, E. A., Lvov, S. D. & Karabatsos, N. California serogroup viruses from mosquitoes collected in the USSR. *Am. J. Trop. Med. Hyg. 2008* **45,** 366–370 (1991).

666. Jentes, E. S. *et al.* Acute arboviral infections in Guinea, West Africa, 2006. *Am. J. Trop. Med. Hyg.* **83,** 388–94 (2010).

667. Madic, J., Huber, D. & Lugovic, B. Serologic survey for selected viral and rickettsial agents of Brown bears *(Ursus arctos)* in Croatia. *J. Wildl. Dis.* **29,** 372–376 (1993).

668. Mickiene, A., Vene, S. & Golovljova, I. Tick-borne encephalitis virus in Lithuania. *Eur. J. Clin. Microbiol. Infect. Dis.* **20,** 886–888 (2001).

669. Lukan, M., Bullova, E. & Petko, B. Climate warming and Tick-borne encephalitis, Slovakia. *Emerg. Infect. Dis.* **16,** 524–6 (2010).

670. Golovljova, I. *et al.* Characterization of Tick-borne encephalitis virus from Estonia. *J. Med. Virol.* **74,** 580–8 (2004).

671. Csángó, P. A., Blakstad, E., Kirtz, G. C., Pedersen, J. E. & Czettel, B. Tick-borne encephalitis in southern Norway. *Emerg. Infect. Dis.* **10,** 533–4 (2004).

672. Rushton, J. O. *et al.* Tick-borne encephalitis virus in horses, Austria, 2011. *Emerg. Infect. Dis.* **19,** (2013).

673. Kaiser, R. Tick-borne encephalitis in southwestern Germany. *Infection* **24,** 398–9 (1996).

674. Takashima, I. Epidemiology of Tick-borne encephalitis in Japan. *Comp. Immunol. Microbiol. Infect. Dis.* **21,** 81–90 (1998).

675. Cisak, E. *et al.* Prevalence of Tick-borne encephalitis virus (TBEV) in samples of raw milk taken randomly from cows, goats, and sheep in eastern Poland. *Ann. Agric. Environ. Med.* **17,** 283–286 (2010).

676. Khasnatinov, M. *et al.* Specific point mutations in the envelope protein of Tick-borne encephalitis virus enhance non-viraemic transmission efficiency in a tick vector. *Int. J. Infect. Dis.* **14,** e372 (2010).

677. Balogh, Z. *et al.* Tick-borne encephalitis outbreak in Hungary due to consumption of raw goat milk. *J. Virol. Methods* **163,** 481–5 (2010).

678. Lindhe, K. E. S., Meldgaard, D. S., Jensen, P. M., Houser, G. A. & Berendt, M. Prevalence of tick-borne encephalitis virus antibodies in dogs from Denmark. *Acta Vet. Scand.* **51,** 56 (2009).

679. Kim, S. Y. *et al.* Molecular evidence for tick-borne encephalitis virus in ticks in South Korea. *Med. Vet. Entomol.* **23,** 15–20 (2009).

680. Lu, Z., Bröker, M. & Liang, G. Tick-borne encephalitis in mainland China. *Vector borne zoonotic Dis.* **8,** 713–20 (2008).

681. Thorin, C. *et al.* Seroprevalence of Lyme Borreliosis and tick-borne encephalitis in workers at risk, in eastern France. *Médecine Mal. Infect.* **38,** 533–42 (2008).

682. Brinkley, C., Nolskog, P., Golovljova, I., Lundkvist, Å. & Bergström, T. Tick-borne encephalitis virus natural foci emerge in western Sweden. *Int. J. Med. Microbiol.* **298,** 73–80 (2008).

683. Daniel, M., Kříž, B., Danielová, V. & Beneš, Č. Sudden increase in Tick-borne encephalitis cases in the Czech Republic, 2006. *Int. J. Med. Microbiol.* **298,** 81–87 (2008).

684. Yaiw, K. C. *et al.* Tioman virus, a Paramyxovirus of bat origin, causes mild disease in pigs and has a predilection for lymphoid tissues. *J. Virol.* **82,** 565–8 (2008).

685. Yaiw, K. C. *et al.* Serological evidence of possible human infection with Tioman virus, a newly described Paramyxovirus of bat origin. *J. Infect. Dis.* **196,** 884–6 (2007).

686. Okamoto, H. *et al.* Species-specific TT viruses and cross-species infection in nonhuman primates. *J. Virol.* **74,** 1132–1139 (2000).

687. Heroldová, M. *et al.* Tula virus in populations of small terrestrial mammals in a rural landscape. *Vector Borne Zoonotic Dis.* **10,** 599–603 (2010).

688. Clement, J., Frans, J. & Van Ranst, M. Human Tula virus infection or rat-bite fever? *Eur. J. Clin. Microbiol. Infect. Dis.* **22,** 332–3; author reply 334–5 (2003).

689. Schultze, D., Lundkvist, A., Blauenstein, U. & Heyman, P. Tula virus infection associated with fever and exanthema after a wild rodent bite. *Eur. J. Clin. Microbiol. Infect. Dis.* **21,** 304–6 (2002).

690. Calzolari, M. *et al.* Evidence of simultaneous circulation of West Nile and Usutu viruses in mosquitoes sampled in Emilia-Romagna region (Italy) in 2009. *PLoS One* **5,** e14324 (2010).

691. Chvala, S., Kolodziejek, J., Nowotny, N. & Weissenböck, H. Pathology and viral distribution in fatal Usutu virus infections of birds from the 2001 and 2002 outbreaks in Austria. *J. Comp. Pathol.* **131,** 176–85 (2004).

692. Weissenböck, H. *et al.* Usutu virus activity in Austria, 2001–2002. *Microbes Infect.* **5,** 1132–1136 (2003).

693. Weissenböck, H. *et al.* Emergence of Usutu virus, an African mosquito-borne Flavivirus of the Japanese Encephalitis virus group, Central Europe. *Emerg. Infect. Dis.* **8,** 652–656 (2002).

694. Manarolla, G. *et al.* Usutu virus in wild birds in northern Italy. *Vet. Microbiol.* **141,** 159–63 (2010).

695. Pecorari, M. *et al.* First human case of Usutu virus neuoinvasive infection, Italy, August-September 2009. *Euro Surveill.* **14,** 5–6 (2009).

696. Cavrini, F. *et al.* Usutu virus infection in a patient who underwent orthotropic liver transplantation, Italy, August - September 2009. *Euro Surveill.* **14,** 1–2 (2009).

697. Lelli, R. *et al.* Serological evidence of Usutu virus occurrence in north-eastern Italy. *Zoonoses Public Health* **55,** 361–7 (2008).

698. Meister, T. *et al.* Serological evidence of continuing high Usutu virus (Flaviviridae) activity and establishment of herd immunity in wild birds in Austria. *Vet. Microbiol.* **127,** 237–48 (2008).

699. Bakonyi, T. *et al.* Emergence of Usutu virus in Hungary. *J. Clin. Microbiol.* **45,** 3870–4 (2007).

700. Chvala, S. *et al.* Monitoring of Usutu virus activity and spread by using dead bird surveillance in Austria, 2003-2005. *Vet. Microbiol.* **122,** 237–45 (2007).

701. Busquets, N., Alba, A., Allepuz, A., Aranda, C. & Ignacio Nuñez, J. Usutu virus sequences in *Culex pipiens* (Diptera: Culicidae), Spain. *Emerg. Infect. Dis.* **14,** 861–3 (2008).

702. Figuerola, J. *et al.* West Nile virus antibodies in wild birds, Morocco, 2008. *Emerg. Infect. Dis.* **15,** (2009).

703. Buckley, A., Dawson, A. & Gould, E. A. Detection of seroconversion to West Nile virus, Usutu virus and Sindbis virus in UK sentinel chickens. *Virol. J.* **3,** 71 (2006).

704. Auguste, A. J. *et al.* Isolation and phylogenetic analysis of Mucambo virus (Venezuelan equine encephalitis complex subtype IIIA) in Trinidad. *Virology* **392,** 123–30 (2009).

705. Hommel, D., Heraud, J. M., Hulin, A. & Talarmin, A. Association of Tonate virus (subtype IIIB of the Venezuelan equine encephalitis complex) with encephalitis in a human. *Clin. Infect. Dis.* **30,** 188–90 (2000).

706. Navarro, J.-C. *et al.* Postepizootic persistence of Venezuelan equine encephalitis virus, Venezuela. *Emerg. Infect. Dis.* **11,** 1907–15 (2005).

707. Estrada-Franco, J. G. *et al.* Venezuelan equine encephalitis virus, southern Mexico. *Emerg. Infect. Dis.* **10,** 2113–21 (2004).

708. Weaver, S. C., Ferro, C., Barrera, R., Boshell, J. & Navarro, J.-C. Venezuelan equine encephalitis. *Annu. Rev. Entomol.* **49,** 141–74 (2004).

709. Aguilar, P. V *et al.* Genetic characterization of Venezuelan equine encephalitis virus from Bolivia, Ecuador and Peru: identification of a new subtype ID lineage. *PLoS Negl. Trop. Dis.* **3,** e514 (2009).

710. Ferro, C. *et al.* Natural enzootic vectors of Venezuelan equine encephalitis virus, Magdalena Valley, Colombia.pdf. *Emerg. Infect. Dis.* **9,** 49–54 (2003).

711. Quiroz, E., Aguilar, P. V, Cisneros, J., Tesh, R. B. & Weaver, S. C. Venezuelan equine encephalitis in Panama: fatal endemic disease and genetic diversity of etiologic viral strains. *PLoS Negl. Trop. Dis.* **3,** e472 (2009).

712. Maness, K. S. C. & Calisher, C. H. Eastern Equine Encephalitis in the United States , 1971 : Past and Prologue. *Curr. Microbiol.* **5,** 311–316 (1981).

713. Lichty, B. D., Power, A. T., Stojdl, D. F. & Bell, J. C. Vesicular stomatitis virus: re-inventing the bullet. *Trends Mol. Med.* **10,** 210–216 (2004).

714. Remmers, L. *et al.* Longitudinal studies in the epidemiology of vesicular stomatitis on Costa Rican dairy farms. *Ann. New York Acad. Scinences* **916,** 417–430 (2000).

715. Hanson, R. P. The natural history of vesicular stomatitis. *Bacteriol. Rev.* **16,** 179–204 (1952).

716. Letchworth, G. J. & Rodriguez, L. L. Review Vesicular Stomatitis. *Vet. J.* **157,** 239–260 (1999).

717. Perez, A. M. *et al.* Spatial and phylogenetic analysis of vesicular stomatitis virus over-wintering in the United States. *Prev. Vet. Med.* **93,** 258–64 (2010).

718. Drolet, B. S., Stuart, M. A. & Derner, J. D. Infection of Melanoplus sanguinipes grasshoppers following ingestion of rangeland plant species harboring Vesicular stomatitis virus. *Appl. Environ. Microbiol.* **75,** 3029–33 (2009).

719. Mead, D. G. *et al.* Black fly involvement in the epidemic transmission of vesicular stomatitis New Jersey virus (Rhabdoviridae: Vesiculovirus). *Vector Borne Zoonotic Dis.* **4,** 351–9 (2004).

720. Rainwater-Lovett, K., Pauszek, S. J., Kelley, W. N. & Rodriguez, L. L. Molecular epidemiology of Vesicular stomatitis New Jersey virus from the 2004-2005 US outbreak indicates a common origin with Mexican strains. *J. Gen. Virol.* **88,** 2042–51 (2007).

721. Rodrı́guez, L. L. Emergence and re-emergence of vesicular stomatitis in the United States. *Virus Res.* **85,** 211–219 (2002).

722. Scherer, C. F. C. *et al.* Vesicular stomatitis New Jersey virus (VSNJV) infects keratinocytes and is restricted to lesion sites and local lymph nodes in the bovine, a natural host. *Vet. Res.* **38,** 375–390 (2007).

723. Diallo, M. *et al.* Mosquito vectors of the 1998-1999 outbreak of Rift Valley Fever and other arboviruses (Bagaza, Sanar, Wesselsbron and West Nile) in Mauritania and Senegal. *Med. Vet. Entomol.* **19,** 119–26 (2005).

724. Mushi, E. Z., Binta, M. G. & Raborokgwe, M. Wesselsbron disease virus associated with abortions in goats in Botswana. *J. Vet. Diagnostic Investig.* **10,** 191–191 (1998).

725. Guilherme, J. M., Gonella-Legall, C., Legall, F., Nakoume, E. & Vincent, J. Seroprevalence of five arboviruses in Zebu cattle in the Central African Republic. *Trans. R. Soc. Trop. Med. Hyg.* **90,** 31–3 (2000).

726. Urray, K. O. M., Ertens, E. M. & Espre, P. D. Review article West Nile virus and its emergence in the United States of America. *Vet. Res.* **41,** (2010).

727. Kramer, L. D., Styer, L. M. & Ebel, G. D. A Global Perspective on the Epidemiology of West Nile Virus. *Annu. Rev. Entomol.* **53,** 61–81 (2008).

728. Chevalier, V. *et al.* Environmental risk factors of West Nile virus infection of horses in the Senegal River basin. *Epidemiol. Infect.* **138,** 1601–9 (2010).

729. Venter, M. & Swanepoel, R. West Nile virus lineage 2 as a cause of zoonotic neurological disease in humans and horses in southern Africa. *Vector borne zoonotic Dis.* **10,** 659–64 (2010).

730. Campbell, G. L., Marfin, A. A., Lanciotti, R. S., Gubler, D. J. & Nile, W. Reviews West Nile virus. *Lancet Infect. Dis.* **2,** 519–529 (2002).

731. America, S. West Nile Virus , Venezuela. *Emerg. Infect. Dis.* **13,** (2007).

732. Abad, R., Enríquez, R., Salcedo, C. & Vázquez, J. A. West Nile Virus in birds, Argentina. *Emerg. Infect. Dis.* **14,** 2006–2008 (2008).

733. Zacks, M. A. & Paessler, S. Encephalitic Alphaviruses. *Vet. Microbiol.* **140,** 281–6 (2010).

734. Forrester, N. L., Kenney, J. L., Deardorff, E., Wang, E. & Weaver, S. C. Western Equine Encephalitis submergence: lack of evidence for a decline in virus virulence. *Virology* **380,** 170–2 (2008).

735. Fulhorst, C. F., Milazzo, M. L., Carroll, D. S., Charrel, R. N. & Bradley, R. D. Natural host relationships and genetic diversity of Whitewater Arroyo virus in southern Texas. *Am. J. Trop. Med. Hyg.* **67,** 114–8 (2002).

736. Fulhorst, C. F. *et al.* Hantavirus and Arenavirus antibodies in persons with occupational rodent exposure, North America. *Emerg. Infect. Dis.* **13,** 532–538 (2007).

737. Fulhorst, C. F. *et al.* Geographic distribution and genetic diversity of Whitewater Arroyo virus in the southwestern United States. *Emerg. Infect. Dis.* **7,** 403–407 (2001).

738. Charrel, R. N., de Lamballerie, X. & Fulhorst, C. F. The Whitewater Arroyo virus: natural evidence for genetic recombination among Tacaribe serocomplex viruses (family Arenaviridae). *Virology* **283,** 161–6 (2001).

739. Milazzo, M. L. *et al.* Antibodies to Tacaribe serocomplex viruses (family Arenaviridae, genus Arenavirus) in cricetid rodents from New Mexico, Texas, and Mexico. *Vector borne zoonotic Dis.* **10,** 629–37 (2010).

740. Inizan, C. C. *et al.* Genetic evidence for a Tacaribe serocomplex virus, Mexico. *Emerg. Infect. Dis.* **16,** 1007–1011 (2010).

741. Forrester, N. L., Kenney, J. L., Deardorff, E., Wang, E. & Weaver, S. C. Western Equine Encephalitis submergence: lack of evidence for a decline in virus virulence. *Virology* **380,** 170–2 (2008).

742. Diaz, H. F. & Mccabe, G. J. A possible connection between the 1878 Yellow Fever epidemic in the southern United States and the 1877 – 78 El Niño episode. *Bull. Am. Meteorol. Soc.* **80,** 21–28 (1999).

743. Monath, T. P. Reviews Yellow fever: an update. *Lancet* **1,** 11–20 (2001).

744. Auguste, A. J. *et al.* Yellow fever virus maintenance in Trinidad and its dispersal throughout the Americas. *J. Virol.* **84,** 9967–77 (2010).

745. J, B. *et al.* Enzootic transmission of Yellow fever virus in Peru. *Emerg. Infect. Dis.* **9,** 926–933 (2003).

746. Bryant, J. E., Holmes, E. C. & Barrett, A. D. T. Out of Africa: a molecular perspective on the introduction of Yellow fever virus into the Americas. *PLoS Pathog.* **3,** e75 (2007).

747. Deng, I. M. *et al.* Ebola haemorrhagic fever in Sudan, 1976. *Bull. World Health Organ.* **56,** 247–270 (1978).

748. Pourrut, X. *et al.* Large serological survey showing cocirculation of Ebola and Marburg viruses in Gabonese bat populations, and a high seroprevalence of both viruses in *Rousettus aegyptiacus*. *BMC Infect. Dis.* **9,** 1–10 (2009).

749. Lahm, S. A., Kombila, M., Swanepoel, R. & Barnes, R. F. W. Morbidity and mortality of wild animals in relation to outbreaks of Ebola haemorrhagic fever in Gabon, 1994-2003. *Trans. R. Soc. Trop. Med. Hyg.* **101,** 64–78 (2007).

750. Leroy, E. M. *et al.* A serological survey of Ebola virus infection in central African nonhuman primates. *J. Infect. Dis.* **190,** 1895–9 (2004).

751. Groseth, A., Feldmann, H. & Strong, J. E. The ecology of Ebola virus. *Trends Microbiol.* **15,** 408–16 (2007).

752. Walsh, P. D., Biek, R. & Real, L. a. Wave-like spread of Ebola Zaire. *PLoS Biol.* **3,** e371 (2005).

753. Allela, L. *et al.* Ebola virus antibody prevalence in dogs and human risk. *Emerg. Infect. Dis.* **11,** 385–90 (2005).

754. Leroy, E. M. *et al.* Multiple Ebola virus transmission events and rapid decline of central African wildlife. *Science* **303,** 387–390 (2004).

755. Hayes, E. B. Zika virus outside Africa. *Emerg. Infect. Dis.* **15,** 1347–1350 (2009).

756. Kool, J. L. *et al.* Zika virus outbreak on Yap Island, Federated States of Micronesia. *N. Engl. J. Med.* **360,** 2536–2543 (2009).

757. Lanciotti, R. S. *et al.* Genetic and serologic properties of Zika virus associated with an epidemic, Yap State, Micronesia, 2007. *Emerg. Infect. Dis.* **14,** 1232–1239 (2008).

758. Fagbami, a H. Zika virus infections in Nigeria: virological and seroepidemiological investigations in Oyo State. *J. Hyg. (Lond).* **83,** 213–9 (1979).

759. Weissenböck, H., Hubálek, Z., Bakonyi, T. & Nowotny, N. Zoonotic mosquito-borne flaviviruses: worldwide presence of agents with proven pathogenicity and potential candidates of future emerging diseases. *Vet. Microbiol.* **140,** 271–80 (2010).

760. Kilbourn, A. M. *et al.* Health evaluation of free-ranging and semi-captive Orangutans *(Pongo pygmaeus pygmaeus)* in Sabah, Malaysia. *J. Wildl. Dis.* **39,** 73–83 (2003).

761. Faye, O. *et al.* One-step RT-PCR for detection of Zika virus. *J. Clin. Virol.* **43,** 96–101 (2008).
